# Endothelial cells-derived SEMA3G suppresses glioblastoma stem cells by inducing c-Myc degradation

**DOI:** 10.1038/s41418-025-01534-3

**Published:** 2025-06-18

**Authors:** Peng-Xiang Min, Li-Li Feng, Yi-Xuan Zhang, Chen-Chen Jiang, Hong-Zhen Zhang, Yan Chen, Kohji Fukunaga, Fang Liu, Yu-Jie Zhang, Takuya Sasaki, Xu Qian, Katsuhisa Horimoto, Jian-Dong Jiang, Ying-Mei Lu, Feng Han

**Affiliations:** 1 Key Laboratory of Modern Toxicology of Ministry of Education; School of Basic Medical Sciences, Nanjing Medical University, Nanjing, China; 2 The Second People’s Hospital of Changzhou, The Third Affiliated Hospital of Nanjing Medical University, Changzhou Medical Center, Nanjing Medical University, Changzhou, China; 3 Medical Basic Research Innovation Center for Cardiovascular and Cerebrovascular Diseases, Ministry of Education; International Joint Laboratory for Drug Target of Critical Illnesses; Jiangsu Key Laboratory of Drug Targets and Translational Medicine for Cardio-cerebrovascular Diseases, School of Pharmacy, Nanjing Medical University, Nanjing, China; 4 Department of CNS Drug Innovation, Graduate School of Pharmaceutical Sciences, Tohoku University, Miyagi, Japan; 5 Department of Pharmacology, Graduate School of Pharmaceutical Sciences, Tohoku University, Sendai, Japan; 6 Department of Neurosurgery of First Affiliated Hospital of Nanjing Medical University, Department of Nutrition and Food Hygiene of School of Public Health, Nanjing Medical University, Nanjing, China; 7 SOCIUM Inc. AIST Waterfront Center Annex 5 F, Aomi 2-4-7, Kotoku, Tokyo, Japan; 8 Saitama Medical University International Medical Center, 1397-1 Yamane, Hidaka, Saitama, Japan; 9 Department of Virology, Institute of Medicinal Biotechnology, Chinese Academy of Medical Sciences and Peking Union Medical College, Beijing, China; 10 Department of Nephrology, the First Affiliated Hospital of Nanjing Medical University, Nanjing, China; 11 Institute of Brain Science, the Affiliated Brain Hospital of Nanjing Medical University, Nanjing, China; 12 The affiliated Huaian No.1 People’s Hospital of Nanjing Medical University, Northern Jiangsu Institute of Clinical Medicine, Huaian, China

**Keywords:** Cancer microenvironment, CNS cancer, Cancer stem cells

## Abstract

The poor prognosis of glioblastoma (GBM) patients is attributed mainly to abundant neovascularization and presence of glioblastoma stem cells (GSCs). GSCs are preferentially localized to the perivascular niche to maintain stemness. However, the effect of abnormal communication between endothelial cells (ECs) and GSCs on GBM progression remains unknown. Here, we reveal that ECs-derived SEMA3G, which is aberrantly expressed in GBM patients, impairs GSCs by inducing c-Myc degradation. SEMA3G activates NRP2/PLXNA1 in a paracrine manner, subsequently inducing the inactivation of Cdc42 and dissociation of Cdc42 and WWP2 in GSCs. Once released, WWP2 interacts with c-Myc and mediates c-Myc degradation via ubiquitination. Genetic deletion of *Sema3G* in ECs accelerates GBM growth, whereas *SEMA3G* overexpression or recombinant SEMA3G protein prolongs the survival of GBM bearing mice. These findings illustrate that ECs play an intrinsic inhibitory role in GSCs stemness via the SMEA3G-c-Myc distal regulation paradigm. Targeting SEMA3G signaling may have promising therapeutic benefits for GBM patients.

## Introduction

Glioblastoma (GBM) is the most common malignant brain tumor in adults with a high mortality rate and limited therapeutic options. One of the most important characteristics of GBM is identified as the extensive aberrant neovascularization [1, 2]. Abnormally formed blood vessels supply the necessary nutrients and oxygen for the rapid progression of tumor tissues [3, 4]. Endothelial cells (ECs) in GBM tissues are pivotal drivers of tumor angiogenesis, primarily through their secretion of proangiogenic factors including vascular endothelial growth factor (VEGF), fibroblast growth factor (FGF), and angiopoietins. These mediators collectively promote tumor neovascularization and invasive progression [5]. Paradoxically, clinical evidence suggests that elevated CXCL10 in subpopulation of glioma-associated vasculature are associated with improved patient outcomes, particularly in those undergoing IL-13Rα2-targeted chimeric antigen receptor (CAR) T-cell therapy [6, 7]. These findings underscore the remarkable heterogeneity of ECs in GBM and suggest that targeted modulation of key EC-derived regulatory factors could disrupt the formation of pro-tumorigenic niches [8, 9].

Glioblastoma stem cells (GSCs) are a subpopulation of neoplastic cells exhibiting stem-like properties within tumor tissues, with the potential to initiate tumor growth, therapeutic resistance, and recurrence [10, 11]. GSCs are often located in a specialized microenvironment that is known as the perivascular niche (PVN), which is formed mainly by ECs. The proximity of GSCs to ECs within the PVN provides support for the maintenance and self-renewal of GSCs [12]. Although the bidirectional interactions between GSCs and ECs favor GBM progression, the sparse abnormal vasculature and reduced endothelial cell numbers in the hypoxic niche provide powerful stimulation for promoting GSC stemness [13, 14]. On the other hand, treatment with bevacizumab (a VEGF inhibitor) has the potential to increase the invasiveness of GBM [15]. These findings indicate the diversity of ECs and the complicated influence on GSCs. Properly targeting the key factors that orchestrate ECs phenotypes may represent a promising therapeutic strategy to disrupt GSC stemness during tumorigenesis.

Class 3 semaphorins (SEMA3s) are a family of secretory proteins that can affect GBM progression by regulating intercellular communication [16]. SEMA3G is an extracellular signaling molecule that is expressed mainly in ECs and has been identified as a prognostic marker in gliomas [17, 18]. The artificial overexpression of SEMA3G in GBM cells was reported to inhibit migration and invasion by suppressing MMP2 activation in vitro [19]. These previous papers indicate the role of aberrant SEMA3G in tumor biology. We previously identified an EC-derived SEMA3G-mediated transcellular remote regulation pattern [20]. Here, we hypothesize that ECs-derived SEMA3G may affect GBM progression by remodeling intercellular crosstalk in a paracrine manner.

As one of the most pivotal stemness markers, c-Myc plays an important role in GBM development and treatment resistance [21]. In the present study, we reveal that ECs-derived SEMA3G effectively protects against GBM by inducing c-Myc degradation, which disrupts the internal driving force of GSC stemness. Interference with the SEMA3G-c-Myc axis may increase the susceptibility to GBM therapy.

## Materials and methods

### Clinical samples

GBM and traumatic brain injury tissue and cerebrospinal fluid (CSF) samples were provided from patients who underwent operation at the Third Affiliated Hospital of Nanjing Medical University (Changzhou, Jiangsu, China). Informed consent was obtained from all participants. Experiments using patient specimens were approved by the institutional review boards of the Third Affiliated Hospital of Nanjing Medical University and conducted in accordance with the Declaration of Helsinki (2013).

### Patient-derived GSCs and cell lines culture

Patient-derived GSC07 and GSC27 cell lines were generously provided by Prof. Xiuxing Wang (Department of Cell Biology, Nanjing Medical University). HEK-293 cells, HEK-293T cells, the GBM cell line T98G were purchased from the American Type Culture Collection (ATCC, VA, USA). Mouse GBM cell line GL261 were purchased from the FUHENG BIOLOGY (Shanghai, China). Short Tandem Repeat (STR) analyses were performed annually on the tumor cells for authentication. Mycoplasma testing with qPCR was performed on supernatants from cell culture at least annually.

Human GSCs were cultured as neurospheres in Neurobasal medium (Gibco, CA, USA) supplemented with B27 supplement without vitamin A (Gibco, CA, USA), Glutamax (Gibco, CA, USA), sodium pyruvate (Gibco, CA, USA), penicillin/streptomycin (Gibco, CA, USA), 20 ng/ml Epidermal growth factor (EGF; R&D Systems, MN, USA), and basic fibroblast growth factor (bFGF; R&D Systems, MN, USA). Human HEK-293, HEK-293T, and T98G cells were cultured in DMEM (Gibco, CA, USA) with 10% fetal bovine serum (FBS; Gibco, CA, USA). Mouse GL261 cells were cultured in DMEM/F12 (1:1) (Gibco, CA, USA) with 10% FBS. All cells were maintained in an incubator at 37°C with 5% carbon dioxide.

The cells were treated with recombinant human SEMA3G protein, Biotin (Sigma-Aldrich, MO, USA), cycloheximide (CHX) (Sigma-Aldrich, MO, USA), MG-132 (Selleck Chemicals, TX. USA), or ML141 (MedChemExpress, NJ, USA) according to the experimental design.

### Mice

*Sema3G*^*f/f*^ mice were generated as our previous description [20]. *Cdh5-Cre* mice were obtained from the Jackson Laboratory (strain no. 006137). *Sema3G*^*f/f*^ mice were crossed with an *Cdh5-Cre* mice to generate *Cdh5-Cre; Sema3G*^*f/f*^ mice. Female BALB/c-nude mice were obtained from the Animal Core Facility at Nanjing Medical University. Mice were housed in an air-conditioned room with a 12-h light/dark cycle and had freely access to water and chow. All experiments and protocols were approved by Nanjing Medical University Animal Experimentation Committee.

### Orthotopic tumor models

Female mice aged 4–6 weeks were randomly assigned to each treatment group. 5 × 10^4^ GL261-Luc cells were implanted into the brain of female *Sema3G*^*f/f*^ or *Cdh5-cre; Sema3G*^*f/f*^ mice at a depth of 3.5 mm (6 mice per group) to establish the intracranial homograft models. 5 × 10^4^ human-derived GSCs (GSC07-Luc) were implanted into the brain of *SEMA3G-*overexpressed or control BALB/c-nude mice (Animal Core Facility of Nanjing Medical University, Nanjing, China) at a depth of 3.5 mm (10 mice per group) to monitor the effect of SEMA3G overexpression.

To evaluate the effect of recombinant SEMA3G protein or ML141, 2.5 × 10^5^ GSC07-Luc cells were implanted into the brains of nude mice to establish a xenograft model (5 mice per group). The mice subsequently received intratumoral injection of SEMA3G protein (1 µg/mouse) or ML141 (200 ng/mouse) every 3 days. Mice were euthanized when they lost more than 20% of their body weight.

### In vivo bioluminescence analysis

To monitor tumor growth in living animals, GL261 and GSC07 cells that were used for animal studies were transduced with firefly luciferase via lentiviral infection. Mice were treated with 120 mg/kg D-luciferin intraperitoneally and anesthetized with isoflurane for imaging analysis. Tumor luciferase images were captured using an IVIS imaging system (IVIS Spectrum; PerkinElmer, MA, USA).

### DNA constructs and oligonucleotides

To knock down WWP2 or PLXNA1 in GSCs, shWWP2, shPLXNA1, or control shRNA oligonucleotides were cloned into the lentiviral vector pCLenti-U6-shRNA, with shRNA expression driven by the U6 promoter. All DNA oligos used are listed in Table S1.

To overexpress WWP2 and ubiquitin in GSCs, the full-length cDNA of the WWP2 or ubiquitin genes was inserted into the pLVX-Puro vector and pLenti-CMV-HA vector, respectively. To overexpress c-Myc, WWP2, NRP2, PLXNA1, UBE4A, UBE4B, UBOX5, FBXL3, FBXW11, Cdc42 or Rac1 in T98G or HEK-293 cells, the full-length or domain deletion mutations cDNA of the c-Myc gene was inserted into the pCMV-N-Flag vector, the full-length cDNA of the WWP2, UBE4A, UBE4B, UBOX5, FBXL3, FBXW11 and PLXNA1 genes was inserted into the pcDNA3.1(+)-Myc-HisA vector, the full-length cDNA of the NRP2 gene was inserted into the pcDNA3.1-HA-C vector, the full-length cDNA of the Cdc42 and Rac1 genes was inserted into the pEGFP-N1 vector. All cDNAs were amplified from the cDNA of GSC07 cells. Site directed mutagenesis was performed on pEGFP-Cdc42 and pEGFP-Rac1 by using PCR.

To produce recombinant SEMA3G protein using HEK-293 cells, the full-length cDNA of the SEMA3G gene was inserted into the pcDNA3.1-C-His vector. To overexpress SEMA3G protein in HUVEC, the full-length cDNA of the SEMA3G gene was inserted into the pLVX-Puro vector. The cDNAs were amplified from the cDNA of HUVEC cells.

For the alkaline phosphatase (AP) ligand binding assays, the coding sequence of human SEAP, with the signal peptide deleted, was amplified by PCR from pCMV-SEAP vector and subcloned into the C-terminal region of SEMA3G in a pcDNA3.1-SEMA3G over expression plasmid.

For proximity labeling, the coding sequence of human NRP2 was amplified by PCR from GSCs and subcloned into the N-terminal region of biotin ligase in a pCMV-miniTurboID-Flag vector.

For bimolecular fluorescence complementation, the coding sequence of human c-Myc subcloned into the N-terminal region of Venus (1-172) in a pBiFC-VN173 vector. The coding sequence of human WWP2 subcloned into the N-terminal region of Venus (155-238) in a pBiFC-VC155 vector. The coding sequence of human wild-type and mutant Cdc42 subcloned into the N-terminal region of Cerulean (1-172) in a pBiFC-VC155 vector. All constructions were verified via commercial Sanger sequencing.

Plasmids were amplified by transforming them into DH5α competent cells (TransGen Biotech, Beijing, China) according to the manufacturer’s instructions. Plasmids were subsequently isolated using the Plasmid Miniprep Kit (Cwbio, Beijing, China) following the manufacturer’s protocol. Plasmid concentrations were determined by measuring absorbance at 260 nm using a Nanodrop One device (Thermo Scientific, MA, USA). All primers used are listed in Table S2.

### Lentiviral transduction and stable cells screening

Lentitirus production was as described previously [22], HEK-293T cells were utilized to generate lentiviral particles by co-transfecting them with the packaging vectors pSPAX2 and pMD2.G using the Polyethylenimine (PEI, Polysciences Inc, PA, USA) transfection reagent in Neurobasal complete medium. HUVECs, GSC07 and GSC27 cells were transfected with the lentivirus for 72 h and then changed to fresh medium. The cells were then screened using puromycin (1 μg/ml) for 14 days to obtain the stable transfected cells.

### Conditional medium (CM) from SEMA3G overexpressing HUVECs

To overexpress SEMA3G protein in HUVEC, the full-length cDNA of the SEMA3G gene was inserted into the pLVX-Puro vector. The SEMA3G overexpressing-HUVECs were cultured in DMEM until they reached 50% confluence. The medium was then replaced with Neurobasal medium, and the cells were incubated for an additional 48 h. The supernatant was collected, centrifuged at 1000 × *g* for 10 min, and filtered through a 0.45 μm sterile filter. The supernatant was then mixed with fresh Neurobasal medium at a 1:2 (v/v) ratio to generate the conditioned medium.

### Enzyme-linked immunosorbent assay (ELISA)

The SEMA3G level in human CSF and conditioned medium was measured using a human Semaphorin 3G ELISA kit (CUSABIO, Wuhan, China) following the manufacturer’s instructions.

### Recombinant SEMA3G protein purification

Recombinant SEMA3G were purified as described in our previous study [20]. Briefly, HEK293T cells were cultured until they reached 90% confluence and then transfected with the pcDNA3.1-SEMA3G-His plasmid using PEI. 6 h later, the culture medium was replaced with Opti-MEM reduced serum medium (Gibco, CA, USA), and sodium butyrate (5 mM) was added 24 h post-transfection. The conditioned supernatants were collected 4–5 days later, and the recombinant SEMA3G proteins were purified using a HisTrap HP column (GE Healthcare, IL, USA) via their histidine tag. Following purification, the proteins were dialyzed, aliquoted, and stored at –80 °C until further use.

### Proliferation and neurospheres formation assay

Cell proliferation experiments were conducted by plating cells of interest at a density of 1000 cells/well in a 96-well plate with five replicates. Cell Counting Kit 8 (Apexbio, TX, USA) was used to measure cell proliferation. The cell viability was calculated with the following formula: cell viability = (absorbance of the treated well)—(absorbance of the blank well). Neurosphere formation was measured by in vitro limiting dilution, as previously reported [23]. Briefly, decreasing numbers of cells per well (100, 50, 10, 5, and 1) were plated into 96-well plates. The presentation and number of neurospheres in each well were recorded seven days after plating. Extreme limiting dilution analysis was performed using software available at http://bioinf.wehi.edu.au/software/elda, as previously described [23, 24].

### Western blotting

Cells were collected and lysed in cell lysis buffer (30 mM Tris-HCl, pH 7.5; 150 mM NaCl; 0.5% NP-40; 50 mM NaF with protease inhibitors) and incubated on ice for 30 min. Lysates were centrifuged at 4 °C for 15 min at 12,000 × *g*, and the supernatant was collected. Protein concentration was determined using the Pierce BCA Protein Assay Kit (ThermoFisher, MA, USA). Equal amounts of protein samples were mixed with SDS Laemmli loading buffer, boiled for 5 min, and subjected to SDS-PAGE using Bis-Tris gels. The proteins were then transferred to PVDF membranes. The membranes were blocked with TBS-T supplemented with 5% non-fat dry milk for 90 min and then incubated with primary antibodies against c-Myc (#18583, Cell Signaling Technology, MA, USA), WWP2 (12197-1-AP, Proteintech, Wuhan, China), Ubiquitin (sc-9133, Santa Cruz Biotechnology, CA, US), PLXNA1 (ab23391, Abcam, MA, USA), PLXNA2 (#3994, Cell Signaling Technology, MA, USA), PLXNA3 (AF4075, R&D Systems), PLXNA4 (#3816, Cell Signaling Technology, MA, USA), PLXND1 (ab28762, Abcam MA, USA), SOX2 (#23064, Cell Signaling Technology MA, USA), Cdc42 (#2466S, Cell Signaling Technology, MA, USA), Rac1 (05-389, Millipore, MA, USA), RhoA (10749-1-AP, Proteintech, Wuhan, China), HA-Tag (81290-1-RR, Proteintech, Wuhan, China), MYC-Tag (60003-2-Ig, Proteintech, Wuhan, China), Flag-Tag (F3165, Sigma-Aldrich, MO, USA), GFP (M20004, ABMART, Shanghai, China) or β-actin (81115-1-RR, Proteintech, Wuhan, China) at 4 °C overnight followed by the HRP-conjugated antibody at room temperature for 2 h. The evaluation of immunoblot was imaged using ECL analysis. For all western blot experiments, the blots were imaged using the Tanon Digital Gel Imaging Analysis System (Tanon Life Science Co.,Ltd., Shanghai, China).

### Co-immunoprecipitation (co-IP)

Cells were collected and lysed in IP buffer (20 mM Tris-HCl, 150 mM NaCl, 1% Triton X-100, 0.5% sodium deoxycholate) containing PMSF and a protease inhibitor cocktail at 4 °C for 30 min. The lysates were then centrifuged at 12,000 × *g* for 15 min at 4 °C and the supernatant was collected. The supernatants were incubated overnight at 4 °C with antibodies against WWP2 (12197-1-AP, Proteintech, Wuhan, China), c-Myc (#18583, Cell Signaling Technology, MA, USA), HA-Tag (81290-1-RR, Proteintech, Wuhan, China), MYC-Tag (60003-2-Ig, Proteintech, Wuhan, China), Flag-Tag (F3165, Sigma-Aldrich, MO, USA), or nonspecific IgG. The immune complexes were captured by incubation with Protein A/G PLUS-agarose beads (sc-2003, Santa Cruz Biotechnology, CA, USA) or magnetic beads (B23201, Selleck Chemicals, TX. USA) at 4 °C for 2 h. The beads were washed six times with IP buffer before being subjected to immunoblotting.

### Pull-down assays

For the detection of active RhoA, equal amounts of total cellular protein were incubated with GST-RBD beads (a gift from Dr. Keith Burridge, Department of Cell and Developmental Biology, University of North Carolina, Chapel Hill, NC) captured on MagneGST Glutathione Particles (Promega, WI, USA) at 4 °C with constant rotation for 30 min. For the detection of active Cdc42 and Rac1, equal amounts of total cellular protein were incubated with GST-CRIB beads (a gift from Dr. James E. Casanova, University of Virginia, VA) captured on MagneGST Glutathione Particles at 4 °C with constant rotation for 30 min. The beads were washed three times with washing buffer (4.2 mM Na_2_HPO_4_, 2 mM KH_2_PO_4_, 140 mM NaCl, and 10 mM KCl, pH 7.2). After this period, the beads were captured using a magnetic stand. Following three washes with ice-cold buffer, the beads were resuspended in 2×SDS sample buffer and subjected to immunoblotting.

### Immunofluorescence staining

The brain sections were washed and fixed with 4% paraformaldehyde for 15 min, then permeabilized with 0.2% Triton X-100 for 15 min. Subsequently, the cells were blocked with 1% BSA at room temperature for 1 h, incubated with primary antibodies against NRP2 (#3366, Cell Signaling Technology, MA, USA) overnight, and then with species-matched secondary antibodies conjugated with Alexa Fluor 488 for 1 h at room temperature. Nuclei were stained with DAPI. Images were acquired using a Zeiss LSM800 confocal microscope (Carl Zeiss, Jena, Germany).

### Hematoxylin and eosin (H&E) staining

The brain sections were washed and fixed in 4% paraformaldehyde for 15 min, followed by rehydration through a graded ethanol series (100%, 95%, 85%, 75%) for 3 min at each concentration. Subsequently, the sections were immersed in distilled water for 2 min. Nuclei were stained with hematoxylin solution for 10 min. After staining, the sections were rinsed under running tap water. Differentiation was performed using Differentiation Solution for 10 s, followed by two rinses with tap water, each lasting 5 min. The sections were then counterstained with eosin solution for 10 s to 2 min. Finally, the sections underwent dehydration, clearing, and sealed sheet. Images were captured using a microscope slide scanner (Carl Zeiss, Jena, Germany), and ImageJ software (NIH, MD, USA) was utilized to quantify the brain and the tumor area.

### Alkaline phosphatase ligand binding assays

HEK-293T cells were transfected with AP-tagged SEMA3G or AP-tag vectors. The corresponding supernatant was collected, filtered, and concentrated using Amicon Ultra Filters (Millipore). The supernatant was then mixed with AP substrate buffer (15 ml of 2 M diethanolamine containing 15 μl of 1 M MgCl_2_ and 100 mg of p-nitrophenyl phosphate, pH 9.8), and the absorbance at 405 nm (optical density units per hour) was measured using a spectrophotometer to indicate AP activity. For binding assays, the brain slices were incubated with AP (2 nM) or AP-SEMA3G (2 nM) for 90 min at room temperature, washed with cold HBAH buffer (20 mM HEPES, 0.1% NaN_3_, 0.5 mg/ml BSA, pH 7.0), and fixed for 5 min in 4% PFA. To inactivate endogenous AP activity, the cells were washed with HBS (20 mM HEPES, 150 mM NaCl, pH 7.0) and incubated at 65 °C for 1 h. Finally, 5-bromo-4-chloro-3-indolyl-phosphate (BCIP)/nitroblue tetrazolium (NBT) (Sangon Biotech, Shanghai, China) was used to analyze the in-situ bindings of the AP fusion protein on the cell surface.

### RNA isolation and quantitative real-time PCR (qPCR)

Total cellular RNA was isolated from cells using the Trizol reagent (Takara, Tokyo, Japan). cDNA was synthesized using the HiScript III RT SuperMix for qPCR Kit (Vazyme, Nanjing, China). QPCR was then performed on an Applied Biosystems StepOnePlus cycler using SYBR-Green PCR Master Mix (Vazyme). All primers used are listed in Table S3.

### Proximity labeling assays

For the proximity labeling experiment, NRP2 and miniTurbo fused expression plasmids were transfected into T98G cells using Lipofectamine 3000 for 48 h. Subsequently, the cells were incubated with 50 μM biotin for 10 min. The samples were gently washed five times with ice-cold PBS. The cell pellets were lysed and then centrifuged at 12,000 × *g* for 15 min at 4 °C. The supernatants were incubated overnight at 4 °C with streptavidin magnetic beads (HY-K0208, MedChemExpress, NJ, USA). The beads were washed six times with PBS before being subjected to immunoblotting.

### Bimolecular fluorescence complementation assays

HEK-293 cells were seeded on sterile glass coverslips and co-transfected with c-Myc and VN173 fusion plasmids, WWP2 and VC155 fusion plasmids, or Cdc42 and CrN173 fusion plasmids using Lipofectamine 3000 reagent (Invitrogen, MA, USA), respectively. After 48 h treatment according to the experimental plan, the cells were washed and fixed with 4% paraformaldehyde for 15 min, then permeabilized with 0.2% Triton X-100 for 5 min. Subsequently, nuclei were stained with DAPI. Images were acquired using a Zeiss LSM800 confocal microscope (Carl Zeiss, German).

### Bioinformatics analysis

For bulk RNA-seq and analysis, GBM datasets were obtained from the Gene Expression Omnibus (GEO) with accession numbers GSE50161, GSE116520, GSE151352, GSE90598, and GSE147352 [25–28]. Differential expression genes (DEGs) were identified using a two-tailed *t* test. Bartlett’s test was applied to assess the homogeneity of variance between groups. If variance homogeneity was violated, the Welch t-test was used instead of the classical *t* test for comparing group means. DEGs with a *P* value < 0.01 were considered significantly different between GBM and No-tumor groups.

GSE162631 were used for the single-cell RNA-seq related analysis, which contained the human GBM and peritumoral surgical tissues from 4 patients with GBM [29]. For quality control, normalization and clustering for single-cell RNA, data processing was conducted using the Seurat R package (v4.3.0). The dataset was filtered to include only genes detected in more than 3 cells, cells with at least 200 genes, unique molecular identifiers (UMIs) less than 20 000, and mitochondrial gene content below 10%. Following this, the data underwent normalization, identification of the top 1000 highly variable genes, and principal component analysis (PCA). The processed data were then clustered using uniform manifold approximation and projection (UMAP).

For cell-type annotation, marker genes were identified for each cluster, and cell types were determined based on common cell markers: B cells (*IGHG1, IGHG3, CD79A*), T cells (*CD3D, CD3E, GZMK*), Mural cells (*RGS5, PDGFRB, NOTCH3*), ECs (*CLDN5, VWF, ABCG2, CAVIN2, CD34*), Tumor cells (*SOX2*), Neutrophils (*IL1R2, CXCR2, FPR2*), Dendritic cells (*HLA-DQA1, HLA-DPB1*), and Macrophages (*MKI67, APOC1, CD163*). Clusters that could not be identified were labeled as “Others”.

For differential gene expression and pathway analysis, DEGs were defined by MAST method in Seurat. GO enrichment analysis for sets of DEGs and GSEA enrichment analysis were performed with clusterProfiler (v4.7.1.3) and GSEABase (v1.66.0) separately.

For cell-cell communication, CellChat (v1.6.1) were used to investigate the molecular interaction networks between different cell types depend on the interactions between signaling ligands, receptors and their cofactor. In this study, 4 glioblastoma samples were selected to create CellChat object.

### Statistical analysis

All data were expressed as mean ± s.e.m. Comparisons between two experimental groups were made using the two-tailed unpaired Student’s *t* test. One- or two-way ANOVAs were used for more than two groups as specified, followed by Tukey’s multiple comparisons tests. For non-normally distributed data, the Mann–Whitney *U* test was used to determine statistical significance. Survival curves were analyzed using Kaplan-Meier analysis. The correlation between gene expression and patient survival was performed through the analysis of different brain tumor datasets downloaded from TCGA GBM brain tumor datasets. *P* values were calculated using the log-rank test. Blinding and randomization were performed in all experiments. A *P* value less than 0.05 was considered significant. All statistical analyses were performed using GraphPad Software.

## Results

### ECs-derived SEMA3G is associated with the prognosis of GBM patients

**Fig. 1:**
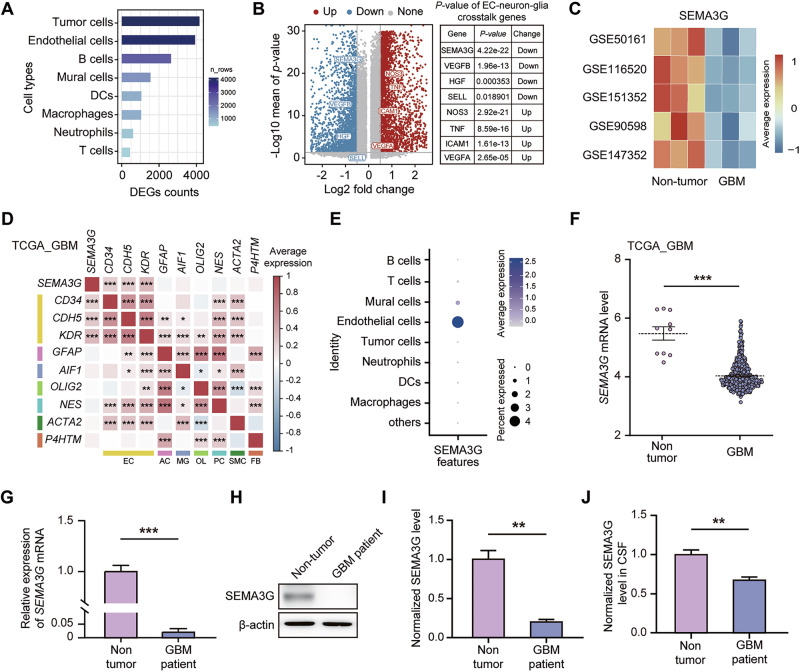
Expression profiles of *SEMA3G* in GBM patients. **A** The differentially expressed genes (DEGs) in various cell types (GSE162631). **B** Volcano plot of DEGs in endothelial cells (GSE162631). **C** Heatmap of *SEMA3G* expression in non-tumor and GBM tissue of different GEO data sets. **D** Bivariate correlation analysis of *SEMA3G* and markers for various types of cells (GSE162631). EC: endothelial cell; AC: astrocyte; MG: microglia; OL: oligodendrocyte; PC: pericyte; SMC: smooth muscle cell; FB: fibroblast. ^*^P < 0.05, ^**^P < 0.01, ^***^P < 0.001, the *P* values were calculated by the *P*earson correlation test. **E** *SEMA3G* expression profile in the various type of cells (GSE162631). **F** The expression level of *SEMA3G* in non-tumor brain (*n* = 10) and GBM tissues (*n* = 521) from the TCGA GBM dataset. ^***^P < 0.001, as determined by the Mann-Whitney *U* test. **G–I** Expression levels of SEMA3G in non-tumor (from traumatic brain injury patients) and GBM tissues from patients. The relative mRNA level of SEMA3G were determined by qPCR (G). The representative bands (**H**) and quantification (**I**) of protein level of SEMA3G as determined by western blot analysis. Data are shown as mean ± s.e.m. *n* = 5, ^**^P < 0.01, ^***^P < 0.001, as determined by two-tailed unpaired *t* test. **J** The protein level of SEMA3G in the cerebrospinal fluid (CSF) of non-tumor (from traumatic brain injury patients) and GBM tissues assessed by ELISA. Data are shown as mean ± s.e.m. *n* = 5, ^**^P < 0.01, as determined by two-tailed unpaired *t*-test.

To identify the intrinsic factors that influence GBM growth, we analyzed single-cell RNA-sequencing (scRNA-seq) datasets (GSE162631) that contains the human GBM and peritumoral surgical tissues from 4 patients with GBM [29] to investigate the differentially expressed genes (DEGs) in various cell types. Tumor cells harbored the greatest number of DEGs, followed closely by ECs (Fig. 1A; Supplementary Fig. 1A, B). Among the DEGs that were detected in ECs, SEMA3G was significantly downregulated, with the lowest *P* value in the well-recognized genes that mediated interactions between ECs and neurons/glia (*P* = 4.22e–22, Fig. 1B). The results from public datasets [25–28] were similar in that the expression of *SEMA3G* was downregulated in GBMs (Fig. 1C). An examination of the TCGA GBM datasets revealed a positive correlation between *SEMA3G* and the markers of ECs (*CD34*, *CDH5*, or *KDR*) but not the markers of other cell types (Fig. 1D). The scRNA-seq dataset of GBM tumors (GSE162631) [29] also revealed the specific expression of *SEMA3G* in ECs (Fig. 1E).

To further determine the potential clinical significance of SEMA3G, we investigated the changes of *SEMA3G* expression in GBM patients. *SEMA3G* exhibited lower mRNA expression levels in GBM tissues than in normal brain tissues (nontumor) in the TCGA GBM database (Fig. 1F). Furthermore, at both mRNA and protein levels, the SEMA3G expression in tumors from GBM patients were markedly lower than those in nontumor brain tissues (Fig. 1G–I). The SEMA3G in the cerebrospinal fluid (CSF) of GBM patients was also significantly lower compared to that of non-tumor patients (Fig. 1J). These data demonstrated the contribution of ECs preferentially expressing SEMA3G to GBM progression.

### ECs-derived SEMA3G suppresses the in vivo GBM growth

**Fig. 2:**
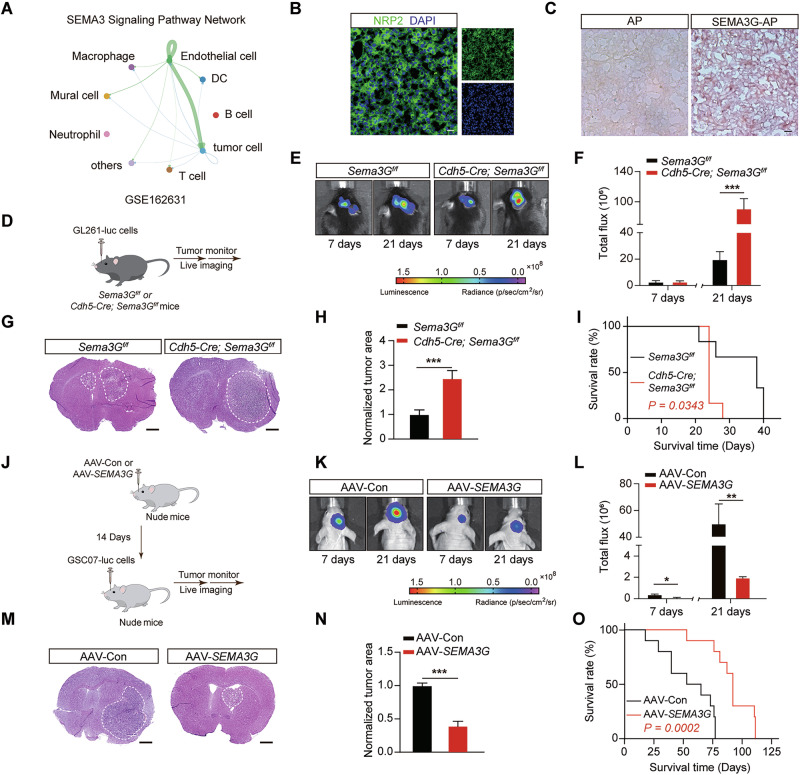
SEMA3G suppresses intracranial GBM growth. **A** Intercellular communication networks of SEMA3s signaling pathways analyzed by CellChat (GSE162631). **B** Immunofluorescent staining of NRP2 in brain sections of tumor-bearing mice. Scale bar, 10 μm. **C** BCIP/NBT color development reveals AP-Ctrl or AP-SEMA3G binding in brain sections of tumor-bearing mice. Scale bar, 5 μm. **D** Schematic diagram of GBM model establishment and monitor using *Sema3G*^*f/f*^ and *Cdh5-cre; Sema3G*^*f/f*^ mice. In vivo bioluminescent image (**E**) and quantification (**F**) of tumor growth on days 7 and 21 post cell implant. Data are shown as mean ± s.e.m. *n* = 6, ^***^P < 0.001. Data were analyzed by two-way ANOVA. Representative images (**G**) and the tumor-to-brain area ratio (**H**) based on the H&E staining mouse brains collected 21 days after transplantation of GL261 cells. Scale bar, 1 mm. Data are shown as mean ± s.e.m. *n* = 6, ^***^P < 0.001, as determined by two-tailed unpaired *t* test. **I** Kaplan-Meier survival curves of the tumor bearing *Sema3G*^*f/f*^ mice and *Cdh5-cre; Sema3G*^*f/f*^ mice. The *P* values were calculated by the log-rank test. *n* = 6. **J** Schematic diagram of overexpressing *SEMA3G* in nude mice and the construction of a GBM model using GSC07-Luc cells. In vivo bioluminescent image (**K**) and quantification (**L**) of tumor growth in GBM bearing mice on days 7 and 21 post cell implant. Data are shown as mean ± s.e.m. *n* = 10, ^*^P < 0.05, ^**^P < 0.01. Data were analyzed by two-tailed unpaired *t* test. **M** H&E staining of mouse brains collec*t*ed 21 days post GSC07 cell transplantation. Scale bar, 1 mm. *n* = 10. **N** The tumor-to-brain area ratio based on the H&E staining (M) was calculated using ImageJ software. Data are shown as mean ± s.e.m. *n* = 10, ^***^P < 0.001, as determined by two-tailed paired *t* test. **O** Kaplan-Meier survival curves of the tumor bearing nude mice. *n* = 10. The *P* values were calculated by the log-rank test. *n* = 10.

Before evaluating the effect of SEMA3G in vivo, we performed CellChat analysis and identify the tumor cells as primary recipients of SEMA3G (Fig. 2A). Then the patient derived GSC07 cells were used to induce orthotopic GBM models in nude mice. The expression of NRP2, the well-known SEMA3G receptor, in tumor areas was confirmed by immunofluorescence analysis (Fig. 2B). Next, we generated alkaline phosphatase-tagged (AP-tagged) recombinant SEMA3G-AP (Supplementary Fig. 2A) and revealed the direct binding of SEMA3G-AP to GBM tumor tissues (Fig. 2C). However, the binding of SEMA3G-AP was attenuated when the brain sections were pre-incubated with an NRP2-neutralizing antibody (Supplementary Fig. 2B). These results provide the basis for the SEMA3G-mediated communication between ECs and tumor cells. To elucidate the role of SEMA3G in GBM growth, EC-specific *Sema3G* knockout mice (*Cdh5-Cre; Sema3G*^*f/f*^) (Supplementary Fig. 3A, B) were used to establish an orthotopic tumor model with syngeneic GL261-Luc cells (Fig. 2D). Our findings indicated that the conditional deletion of *Sema3G* in ECs promoted tumor growth (Fig. 2E–H) and accelerated mouse death (Fig. 2I). On the other hand, we injected *SEMA3G*-overexpressing adeno-associated viruses (AAVs) (Supplementary Fig. 3C) into the striatum of nude mice. Two weeks later, the mRNA and protein levels of SEMA3G in the striatum were significantly elevated compared to control AAVs treat mice (Supplementary Fig. 3D, E). Then, the SEMA3G-overexpressed mice were orthotopically injected with GSC07-Luc cells to establish tumor models (Fig. 2J). Notably, SEMA3G overexpression in the tumor microenvironment resulted in a reduction in tumor size (Fig. 2K–N) and a marked increase in survival duration (Fig. 2O). Furthermore, GSC07-Luc cells were implanted into the brains of nude mice to initiate tumor growth. Three days later, the SEMA3G recombinant protein (Supplementary Fig. 3F, G, 1 µg/mouse) was administered via intratumoral injection (once every 3 days) (Supplementary Fig. 4A). Similarly, recombinant SEMA3G significantly suppressed the tumor growth (Supplementary Fig. 4B–E) and prolonged the survival time (Supplementary Fig. 4F). These data revealed the protective effect of EC-derived SEMA3G on GBM model mice.

### SEMA3G impairs GSC stemness

**Fig. 3:**
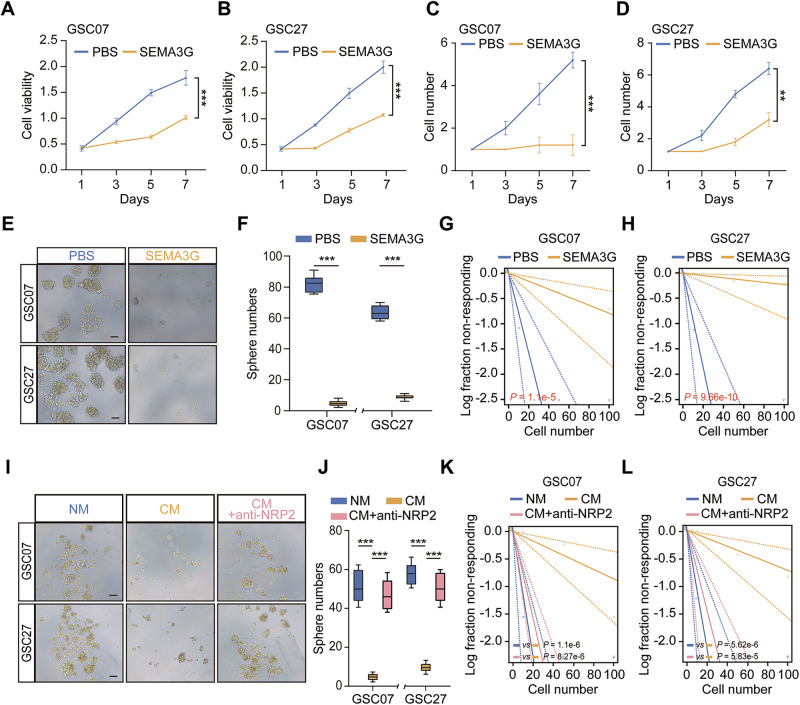
SEMA3G inhibits GSCs growth and self-renewal. GSC07 (**A**) or GSC27 (**B**) cells were incubated with recombinant hSEMA3G protein (200 ng/ml) for the indicated days. Cell viability was measured by CCK8. Data are shown as mean ± s.e.m. ^***^P < 0.001. Data were analyzed by two-way ANOVA. GSC07 (**C**) or GSC27 (**D**) cells were incubated with recombinant hSEMA3G protein (200 ng/ml) for the indicated days. The sphere formation was determined by cell counting. Data are shown as mean ± s.e.m. ^**^P < 0.01, ^***^P < 0.001. Data were analyzed by two-way ANOVA. Representative images (**E**) and quantification (**F**) of neurospheres formed by GSC07 and GSC27 cells treated with 200 ng/ml hSEMA3G for 7 days. Scale bar, 100 μm (**E**). The number of neurospheres was calculated. Data are shown as mean ± s.e.m. ^***^P < 0.001. Data were analyzed two-tailed unpaired *t* test. Sphere formation in GSC07 (**G**) and GSC27 (**H**) cells incubated with recombinant hSEMA3G protein (200 ng/ml) for 7 days by extreme limiting dilution assays. Representative images (**I**) and quantification (**J**) of neurospheres formed by GSC07 and GSC27 cells treated with CM or CM combination with NRP2 neutralizing antibodies for 7 days. Scale bar, 100 μm (**I**). The number of neurospheres was calculated. Data are shown as mean ± s.e.m. ^***^P < 0.001. Data were analyzed by one-way ANOVA. Sphere formation in GSC07 (**K**) and GSC27 (**L**) cells incubated with CM or combination with NRP2 neutralizing antibodies for the 7 days by extreme limiting dilution assays. Scale bar, 100 μm. Data represent the mean ± s.e.m. All data were collected from three independent experiments.

To explore the functional features of SEMA3G, we evaluate the effect of recombinant human SEMA3G protein on GSC, the close interactors with EC. SEMA3G caused a time-dependent reduction in the viability (Fig. 3A, B) and self-renewal (Fig. 3C, D) of both GSC07 and GSC27 cells, concomitantly inhibiting their sphere-forming ability (Fig. 3E, F). SEMA3G also inhibited the proliferation (Supplementary Fig. 5A) and motility of GL261 mouse GBM cells (Supplementary Fig. 5B, C). Data from limiting dilution assays revealed impaired stemness after exposure to recombinant SEMA3G (Fig. 3G, H). To gain insight into the role of ECs derived SEMA3G on GSC, we constructed SEMA3G-overexpressing HUVECs using lentivirus (Supplementary Fig. 6A) and confirmed the expression and secretion of SEMA3G in the medium by ELISA analysis (Supplementary Fig. 6B). Subsequently, the conditional medium (CM) was collected and co-cultured with GSC07 and GSC27 cells, which resulted in the suppressed stemness (Fig. 3I–L). However, the stemness inhibition induced by SEMA3G-overexpressing HUVECs-derived CM was significantly rescued in the presence of NRP2 neutralizing antibody (Fig. 3I–L). These results demonstrated that SEMA3G was mainly involved in the interaction between ECs and GSCs.

### SEMA3G promotes c-Myc degradation

**Fig. 4:**
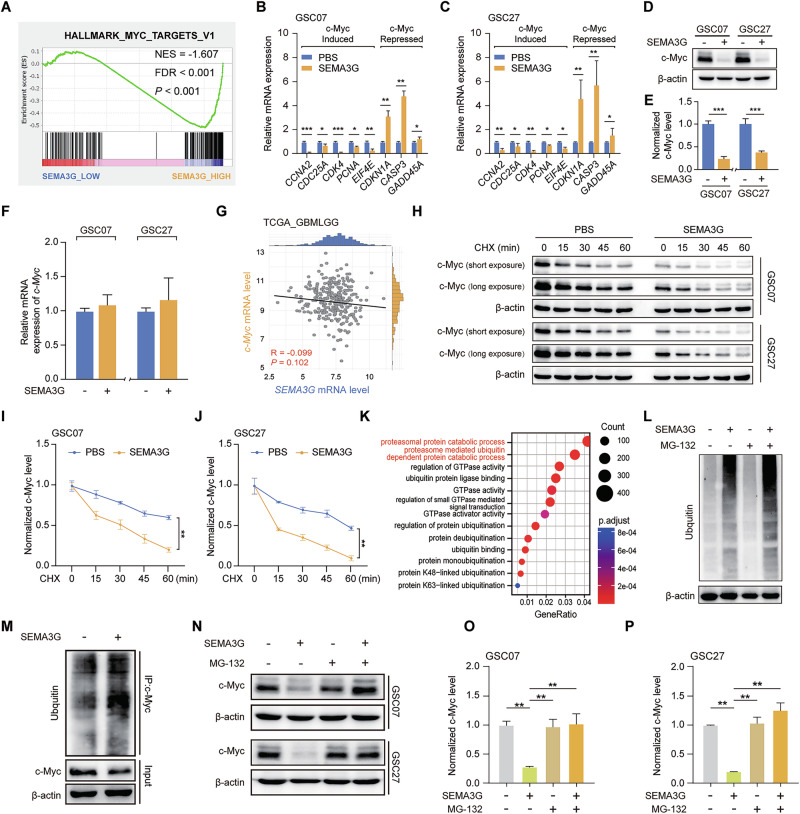
SEMA3G reduces c-Myc protein stability in GSCs. **A** Gene signatures enrichment of c-Myc targets in SEMA3G expressed GBM specimens. mRNA levels of c-Myc target genes in GSC07 (**B**) and GSC27 (**C**) cells treated with or without recombinant hSEMA3G (200 ng/ml) for 72 h. Data are shown as mean ± s.e.m. ^*^P < 0.05, ^**^P < 0.01, ^***^P < 0.001. Data were analyzed by two-way ANOVA. The representative bands (**D**) and quantification (**E**) of c-Myc in GSC07 and GSC27 cells treated with or without recombinant hSEMA3G (200 ng/ml) for 72 h, as determined by western blot analysis. Data are shown as mean ± s.e.m. ^***^P < 0.001. Data were analyzed by two-tailed unpaired *t* test. **F** mRNA levels of c-Myc in GSC07 and GSC27 cells treated with or without recombinant hSEMA3G (200 ng/ml) for 72 h. Data are shown as mean ± s.e.m. Data were analyzed by two-tailed unpaired *t* test. **G** Correlation of *SEMA3G* and *c-Myc* a*t* transcriptional level in GBM specimens from TCGA_GBMLGG dataset. The *P* values were calculated by the Pearson correlation test. **H** The representative bands of c-Myc in GSC07 and GSC27 cells treated with or without recombinant hSEMA3G (200 ng/ml) for 72 h, followed by CHX (10 µg/ml) for various time point. **I**, **J** Data summary of (**H**) are shown as mean ± s.e.m. ^**^P < 0.01. Data were analyzed by two-way ANOVA. **K** GO terms analysis of the DEGs between GBM datasets (GSE162631) with different *SEAM3G* expression levels. **L** The representative bands of ubiquitination in GSC07 cells treated with SEMA3G (200 ng/ml) for 66 h, followed by MG132 (10 μmol/l) or the same volume DMSO for an additional 6 h. **M** The representative bands of the ubiquitination level of c-Myc in GSC07 cells treated with SEMA3G (200 ng/ml) for 72 h. The representative bands (**N**) and quantification (**O**, **P**) of c-Myc in GSC07 and GSC27 incubated with SEMA3G (200 ng/ml) for 66 h, followed by MG132 (10 μmol/l) for an additional 6 h. Data are shown as mean ± s.e.m. ^**^P < 0.01. Data were analyzed by one-way ANOVA. All the western blot bands represent one of the three independent experiments.

To gain insight into the molecular mechanism underlying SEMA3G activity, we determined the effect of SEMA3G on well-known regulators of GSC stemness, including SOX2 and c-Myc. Data from GSEA revealed that the gene signatures of c-Myc targets were enriched in patients who expressed low levels of *SEMA3G* in the GSE162631 dataset (Fig. 4A). In contrast, SOX2 barely fluctuated in patients with aberrant *SEMA3G* expression (Supplementary Fig. 7A, B) and in SEMA3G-treated GSCs (Supplementary Fig. 7C). These results suggested that SEMA3G modulated mainly the c-Myc signal. Treatment of GSC07 and GSC27 cells with the recombinant SEMA3G protein downregulated the expression of a panel of genes whose expression is triggered by c-Myc, and upregulated genes whose expression is inhibited by c-Myc (Fig. 4B, C). However, treatment with SEMA3G markedly decreased c-Myc protein levels in GSCs (Fig. 4D, E) without altering c-Myc mRNA levels (Fig. 4F). No positive correlations between *SEMA3G* and *c-Myc* mRNA levels were observed (Fig. 4G). These results indicated that SEMA3G influenced the protein level of c-Myc rather than affecting its transcription.

After incubation with the recombinant SEMA3G protein, the half-life of the c-Myc protein in GSC07 and GSC27 cells was significantly decreased (Fig. 4H–J). To precisely examine the effect of SEMA3G on c-Myc protein stability, we divided patients in the GSE162631 dataset into two cohorts based on their *SEMA3G* expression level and performed GO analysis. Our data revealed the key involvement of the proteasomal protein catabolic process and the proteasome-mediated ubiquitin-dependent catabolic process (Fig. 4K). Indeed, SEMA3G induced a notable increase in the overall intracellular ubiquitin level (Fig. 4L) and specifically increased c-Myc ubiquitination (Fig. 4M). When GSC07 and GSC27 cells were treated with SEMA3G and subsequently with MG-132 to inhibit proteasome function, the decrease in c-Myc protein levels was reversed (Fig. 4N–P). Taken together, these data demonstrated that SEMA3G induced ubiquitin-proteasome-dependent c-Myc degradation.

### The E3 ubiquitin ligase WWP2 regulates c-Myc degradation in GSCs

**Fig. 5:**
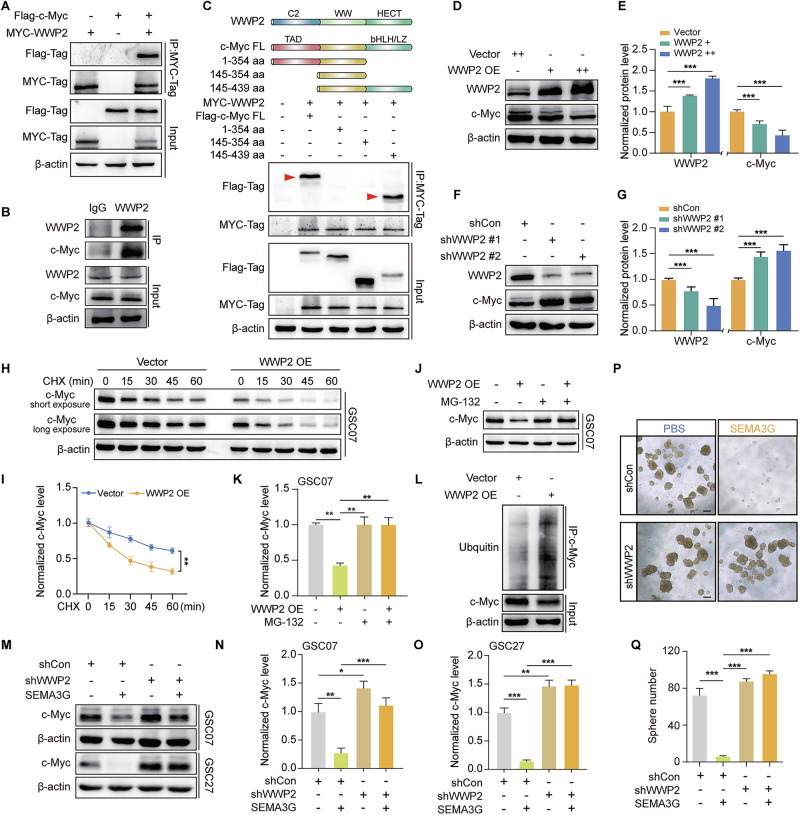
SEMA3G promotes c-Myc degradation in a WWP2-dependent manner. **A** HEK-293 cells were transfected with Flag-tagged c-Myc and/or MYC-tagged WWP2 for 48 h. The interaction of c-Myc and WWP2 were detected using Co-immunoprecipitation. **B** The binding of endogenous WWP2 to c-Myc in GSC07 cells by co-immunoprecipitation analysis. **C** HEK-293 cells were transfected with Flag-tagged c-Myc domains and MYC-tagged WWP2 for 48 h. The interaction of c-Myc and WWP2 were detected using co-immunoprecipitation. **D** The overexpressed GSC07 cells (+) were transfected with WWP2-overexpresson lentivirus again (++) to induced the higher expression of WWP2. The protein level of c-Myc in the GSC07 cells with various WWP2 overexpression was determined by western blot. **E** Data summary of (D) are shown as mean ± s.e.m. ^***^P < 0.001. Data were analyzed by one-way ANOVA. **F** GSC07 cells were transfected with various WWP2 silence lentivirus for 72 h and screened using puromycin (1 μg/ml) to induced the stable knockdown cell. The protein level of c-Myc was determined by western blot. **G** Data summary of (**F**) are shown as mean ± s.e.m. ^***^P < 0.001. Data were analyzed by one-way ANOVA. **H** The WWP2 overexpressed GSC07 cells were treated with CHX (10 µg/ml) for the indicated time points. The protein level of c-Myc was determined using western blot. **I** Data summary of (**H**) are shown as mean ± s.e.m. ^**^P < 0.01. Data were analyzed by two-way ANOVA. **J** The WWP2 overexpressed GSC07 cells were treated with MG132 (μmol/l) for 6 h. The protein level of c-Myc was determined using western blot. **K** Data summary of (J) are shown as mean ± s.e.m. ^**^P < 0.01. Data were analyzed by one-way ANOVA. **L** The ubiquitination level of c-Myc in WWP2 overexpressed GSC07 was determined by co-immunoprecipitation analysis. **M** The WWP2 knockdown GSC07 and GSC27 cells were treated with or without SEMA3G for 72 h. The protein level of c-Myc was determined using western blot. **N, O** Data summary of (M) are shown as mean ± s.e.m. ^**^P < 0.01, ^***^P < 0.001. Data were analyzed by one-way ANOVA. Representative images (**P**) and the number (**Q**) of neurospheres of WWP2 knockdown GSC07 that were treated with or without SEMA3G (200 ng/ml) for 7 days. Images of neurospheres were captured using microscope. Scale bar, 100 μm. Data are shown as mean ± s.e.m. ^***^P < 0.001. Data were analyzed by one-way ANOVA. All the western blot bands represent one of the three independent experiments.

Owing to the critical role of E3 ligases in ubiquitin-proteasome pathway-mediated protein degradation, we predicted E3 ligases that are associated with c-Myc via Ubibrowser 2.0 [30]. Among top six proteins (scores above 0.9) (Supplementary Fig. 8A), UBOX5 and WWP2 were predicted to be related with the survival probability of GBM patients (Supplementary Fig. 8B–G), suggesting their potential involvement in GBM progression. Then, we used the STRING database to analysis the interactions between these candidates and c-Myc [31]. Only WWP2 was predicted to interact with c-Myc when we set the interaction score threshold to > 0.4 (Supplementary Fig. 8H). We also constructed the MYC-tagged predicted E3 ligases and Flag-tagged c-Myc and co-transfected into HEK-293 cells. Immunoprecipitation with a MYC-specific antibody revealed the interaction between WWP2 and c-Myc (Fig. 5A), while other proteins (UBE4A, UBE4B, UBOX5, FBXL3, and FBXW11) weren’t detected (Supplementary Fig. 8I). Interaction between endogenous c-Myc and WWP2 in GSC07 cells was also confirmed via a co-IP assay (Fig. 5B). We subsequently mapped the domains of c-Myc and found that the bHLH/LZ domain located at the C-terminus of c-Myc (amino acids (aa) 354-439) was crucial for its interaction with WWP2 (Fig. 5C). Based on these observations, we focused on the detailed functions of WWP2 on the degradation of c-Myc. Data showed that WWP2 overexpression in GSC07 cells reduced the protein level of c-Myc (Fig. 5D, E), whereas WWP2 silencing resulted in a notable increase in c-Myc (Fig. 5F, G). The protein stability of c-Myc was impaired by WWP2 overexpression, as determined by the shorter half-life of c-Myc in the presence of CHX (Fig. 5H, I). However, WWP2 overexpression failed to alter the c-Myc level when the proteasome was inhibited by MG132 (10 μmol/l) (Fig. 5J, K). The increased ubiquitination of c-Myc under WWP2 overexpression circumstance further confirmed the involvement of WWP2 in c-Myc degradation (Fig. 5L).

More importantly, the SEMA3G-mediated degradation of c-Myc was effectively reversed in WWP2-silenced GSC07 and GSC27 cells (Fig. 5M–O). As a result, the disruption of the sphere-forming capacity of GSC07 cells induced by SEMA3G was significantly impaired by WWP2 knockdown (Fig. 5P, Q). These findings identified WWP2 as the key E3 ligases responsible for SEMA3G-induced ubiquitin-proteasomal degradation of c-Myc.

### SEMA3G engages NRP2/PLXNA1 to inactive Cdc42 in GSCs

**Fig. 6:**
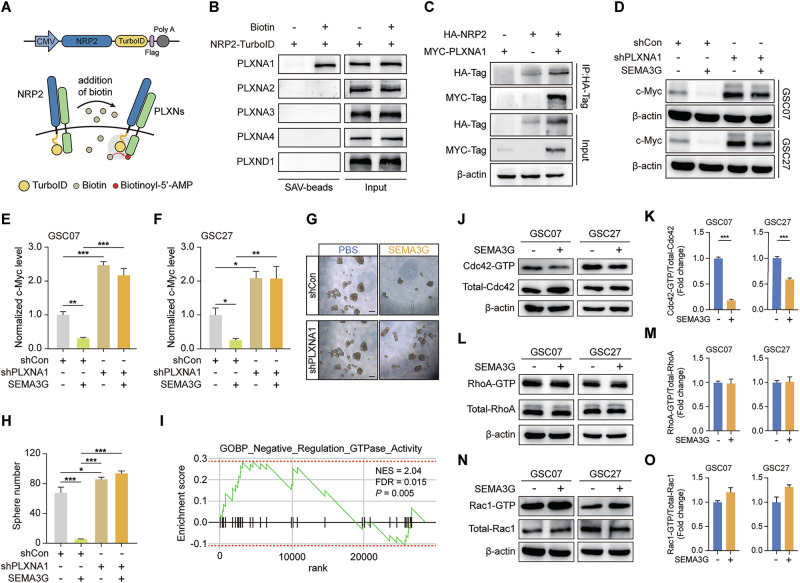
SEMA3G inactivates Cdc42 through NRP2/PLXNA1 in GSCs. **A** Schematic illustration of the TurboID-tagged plasmid constructure and proximity labeling experiment. **B** PLXNA1, PLXNA2, PLXNA3, PLXNA4 and PLXND1 protein levels in whole cell lysates (input) or streptavidin bead–enriched protein samples from T98G cells transfected with NRP2-TurboID construct and treated with or without biotin. **C** HEK-293 cells were transfected with HA-tagged NRP2 and/or MYC-tagged PLXNA1. The interaction was determined using co-immunoprecipitation. **D** The PLXNA1 knockdown and control GSC07 and GSC27 cells treated with or without SEMA3G (200 ng/ml) for 72 h. The protein level of c-Myc was determined using western blot. **E**, **F** Data summary of (**D**) are shown as mean ± s.e.m. ^*^P < 0.05, ^**^P < 0.01, ^***^P < 0.001. Data were analyzed by one-way ANOVA. **G** PLXNA1 knockdown GSC07 cells were treated with or without SEMA3G (200 ng/ml) for 7 days. Images of neurospheres were captured using microscope. Scale bar, 100 μm. **H** Data summary of (**G**) are shown as mean ± s.e.m. ^***^P < 0.001. Data were analyzed by one-way ANOVA. **I** The gene signatures of negative regulation GTPase enrichment analysis. **J–O** GSC07 and GSC27 cells were treated with or without SEMA3G (200 ng/ml) for 72 h. the activation of Cdc42 (**J**), RhoA (**L**), Rac1 (**N**) were determined using GST pulldown assay. **K**, **M**, **O** Data summary of (**J**, **L**, **N**) are shown as mean ± s.e.m. ^***^P < 0.001. Data were analyzed by two-tailed paired *t* test. All the western blot bands represent one of the *t*hree independent experiments.

SEMA3G specifically binds to NRP2 but not NRP1 [32, 33]. However, NRP2 possesses a short intracellular domain, necessitating the formation of a complex coreceptor with PLXNs to facilitate the transmission of SEMA3G signals [34]. We conducted TurboID proximity labeling assays to identify NRP2-associated coreceptors (Fig. 6A). Among the PLXNAs family and PLXND1, PLXNA1 was identified as the coreceptor of NRP2 (Fig. 6B), which was further verified via a co-IP assay (Fig. 6C). Data from a public database also revealed positive correlations between *NRP2* and *PLXNA1* mRNA levels in GBM (Supplementary Fig. 9A). However, the Kaplan-Meier survival analysis for PLXNA1 expression based on TCGA GBM patient database revealed the poor prognosis of high expression patient population (Supplementary Fig. 9B). Our data showed that silencing of *PLXNA1* in GSC07 and GSC27 cells resulted in elevated c-Myc protein levels (Fig. 6D–F). Furthermore, the SEMA3G-induced decrease in c-Myc expression was reversed in *PLXNA1*-silenced cells (Fig. 6D–F). The sphere formation of *PLXNA1*-silenced GSC07 cells was also barely suppressed by SEMA3G (Fig. 6G, H). We further conducted a biological process analysis via GO to gain insight into the downstream signaling pathways of SEMA3G-NRP2/PLXNA1. The results suggested a close association between the expression level of SEMA3G and the negative regulation of GTPase activity within the GSE162631 dataset (Fig. 6I). Active-Cdc42 (Cdc42-GTP) was markedly suppressed in SEMA3G stimulated GSC07 and GSC27 cells (Fig. 6J, K), whereas RhoA-GTP were barely influenced by SEMA3G treatment (Fig. 6L, M). The level of Rac1-GTP demonstrated a slight increase but without statistical significance (Fig. 6N, O). More importantly, even in the presence of Rac1 activation, there was almost no significant effect on c-Myc protein levels and its ubiquitination (Supplementary Fig. 10A). Collectively, these results indicated that SEMA3G suppressed GSC stemness in an NRP2/PLXNA1 dependent manner.

### Cdc42 acts as a molecular switch to regulate WWP2-mediated c-Myc degradation

**Fig. 7:**
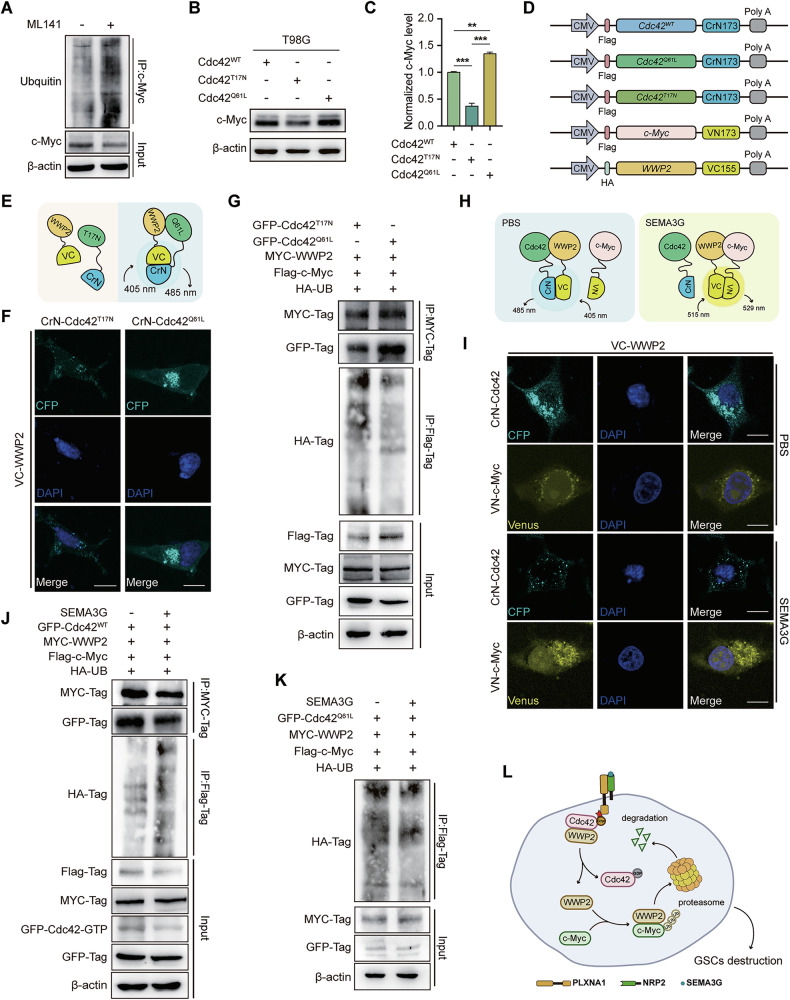
Cdc42 acts as a molecular switch regulating c-Myc degradation by WWP2. **A** The ubiquitination level of c-Myc in GSC07 cells treated with or without ML141 (10 μmol/l) for 24 h. **B**, **C** c-Myc level in T98G cells transfected with GFP-Tagged Cdc42^WT^, Cdc42^T17N^ or Cdc42^Q61L^. **D** Schematic diagram depicting the plasmid construction for bimolecular fluorescence complementation assays. **E** Schematic illustration of the bimolecular fluorescence complementation assay used to assess the interaction between Cdc42 and WWP2**. F** HEK-293 cells were transfected with CrN173-tagged Cdc42^T17N^ or Cdc42^Q61L^, and VC155-tagged WWP2 for 48 h. CFP signal that represents Cdc42 binding to WWP2 were determined using confocal. Scale bar, 5 μm. **G** HEK-293 cells were transfected with Flag-tagged c-Myc, HA-tagged ubiquitin, MYC-tagged WWP2 and GFP-tagged Cdc42^T17N^ or Cdc42^Q61L^ for 48 h. The ubiquitin level of c-Myc and the binding of Cdc42 to WWP2 were determined by co-immunoprecipitation. **H** Schematic illustration of the bimolecular fluorescence complementation assay used to assess the interaction between WWP2 and Cdc42 or c-Myc. **I** HEK-293 cells were incubated with or without SEMA3G (200 ng/ml) for 48 h after co-transfected with CrN173-tagged Cdc42^WT^ or VN173-tagged c-Myc and VC155-tagged WWP2 for 12 h. CFP signal that represents Cdc42 binding to WWP2 and Venus signal that represents WWP2 binding to c-Myc were captured using confocal. Scale bar, 5 μm. **J** HEK-293 cells that were co-transfected with GFP-tagged Cdc42^WT^, MYC-tagged WWP2, Flag-tagged c-Myc, and HA-tagged ubiquitin (UB) for 12 h, followed by the treatment with or without SEMA3G (200 ng/ml) for 48 h. The binding of Cdc42^WT^ to WWP2 and the ubiquitination level of c-Myc were determined by co-immunoprecipitation. **K** HEK-293 cells were co-transfected with GFP-tagged Cdc42^Q61L^ and MYC-tagged WWP2, Flag-tagged c-MYC, and HA-tagged ubiquitin (UB) for 12 h, followed by the treatment with or without SEMA3G (200 ng/ml) for 48 h. The protein level of c-Myc and ubiquitination level of c-Myc were determined by co-immunoprecipitation. **L** Schematic diagram illustrating the potential mechanism by which Cdc42 regulates WWP2 binding to c-Myc and promotes c-Myc degradation. All the western blot bands represent one of the three independent experiments.

To assess the impact of Cdc42 activity on c-Myc protein stability, we utilized a specific small molecule inhibitor of Cdc42, ML141, and found that suppressing Cdc42 activity reduced c-Myc protein levels but also increased the levels of ubiquitinated c-Myc GSC07 cells (Fig. 7A, Supplementary Fig. 10B). Consistently, the self-renewal and sphere-forming abilities of GSC07 and GSC27 cells were significantly inhibited by ML141 (Supplementary Fig. 10C–F). To further evaluate the therapeutic potential of ML141, we conducted in vivo studies using GBM-bearing nude mice via intratumoral administration. ML141 significantly attenuated tumor progression, as evidenced by reduced tumor growth kinetics (Supplementary Fig. 10G, H), decreased tumor burden (Supplementary Fig. 10I, J), and prolonged survival in the murine models (Supplementary Fig. 10K). These findings strongly suggest that Cdc42 activity plays a critical role in maintaining GSC stemness and driving GBM progression, further supporting the therapeutic targeting of Cdc42 in GBM treatment.

Next, we generated constitutively active Cdc42 (Cdc42^Q61L^) and dominant negative Cdc42 (Cdc42^T17N^) proteins to mimic the absence and presence of SEMA3G, respectively, and further used these systems to deeply explore the specific mechanism underlying Cdc42 activity in WWP2-mediated c-Myc degradation. Cdc42^T17N^ reduced the protein level of c-Myc via increased ubiquitination, whereas Cdc42^Q61L^ markedly inhibited c-Myc degradation (Fig. 7B, C; Supplementary Fig. 10L). *Cdc42* and *c-Myc* mRNA levels were positively correlated in the CGGA dataset (Supplementary Fig. 10M). To further elaborate the dynamic interactions and intricate regulatory patterns, we established various forms of Cdc42 with CrN173, c-Myc with VN173, and WWP2 with VC155 (Fig. 7D) and performed bimolecular fluorescence complementation analysis (Fig. 7E). The results revealed that constitutively active Cdc42 (Cdc42^Q61L^) and WWP2 presented stronger fluorescence signals, whereas dominant negative Cdc42 (Cdc42^T17N^) and WWP2 presented only minimal signals in the absence of SEMA3G (Fig. 7F). Data from co-IP assays also revealed a suppressed interaction between Cdc42^T17N^ and WWP2 and increased levels of ubiquitous c-Myc when co-transfected with Cdc42^T17N^ (Fig. 7G). Similarly, the data from Figs. 7H, I further supported that WWP2 exhibits a stronger binding affinity for Cdc42 compared to c-Myc in the absence of SEMA3G. However, upon SEMA3G treatment, WWP2 dissociated from the inactivated form of Cdc42 and shifted its binding preference towards c-Myc (Fig. 7I, the bottom panel).

To further investigate the impact of SEMA3G on Cdc42 activity and the dynamic interactions between Cdc42 and WWP2, a multiple plasmid co-transfection assay was conducted. As shown in Fig. 7J, SEMA3G decreased the level of the Cdc42-GTP, attenuated Cdc42 and WWP2 interaction, but increased ubiquitination of c-Myc with reduced c-Myc protein level. Nevertheless, SEMA3G failed to alter the ubiquitination of c-Myc in the presence of Cdc42^Q61L^, which is a mutant that cannot be inactivated (Fig. 7K). These data suggested that SEMA3G induced the dissociation of WWP2 from Cdc42 by promoting Cdc42 inactivation, subsequently facilitating the binding of WWP2 to c-Myc. This process ultimately accelerated the ubiquitination and degradation of c-Myc (Fig. 7L).

## Discussion

As the main structural component of GSC habitats, the functional phenotype of ECs is constantly remodeled to provide a microenvironment that favors the maintenance of GSC [3, 12, 35]. However, emerging evidence also reveals the highly heterogeneous role of ECs in GBM including the induction of vascular normalization and the promotion of antitumor immune responses [36–38]. Our study identifies SEMA3G as an important EC-derived signal that directly impairs GSC stemness and inhibits GSC-originated tumor growth in mice. Our research further reveals that SEMA3G orchestrates WWP2-mediated c-Myc degradation through the NRP2/PLXNA1/Cdc42 axis in GSCs. The interaction paradigm between ECs and GSCs mediated by SEMA3G may skew the microenvironment toward a less suitable niche for GSCs.

Here, we provide the first evidence of decreased SEMA3G expression in GBM patients. Our preclinical data indicates that *Sema3G* deficiency in ECs promotes GBM growth in mice. As the primary structural components of the PVN, ECs contribute to maintain the stemness of the GSC through secretion of bioactive factors [39]. Strikingly, our experimental data demonstrates that CM from SEMA3G-overexpressing HUVECs significantly suppresses GSC stemness. This inhibitory effect is likely mediated by the concerted activity of SEMA3G and other bioactive factors secreted by HUVECs. Pharmacological blockade of the SEMA3G receptor NRP2 substantially reverses stemness suppression, confirming the SEMA3G-mediated ECs-GSC crosstalk.

As a secreted protein, SEMA3G must interact with specific receptors to exert its pharmacological effects on recipient cells. NRP2/PLXNA4 have been identified as the transcellular receptors of SEMA3G to regulate the synaptic structure and plasticity of pyramidal neurons in the hippocampus [20]. Additionally, NRP2 and PLXND1 are involved in modulating physiological vascular remodeling in ischemic retinopathies[22]. Here, we identify the paracrine mechanism fulfilled by the ECs-derived SEMA3G and its interaction with NRP2/PLXNA1 receptors in GSCs, which provides critical insights into the role of SEMA3G in facilitating remote communication in GBM. Furthermore, the cell-type-specific receptor binding pattern of SEMA3G may contribute to the development of precision therapy strategies for GBM.

The functional role of PLXNA1 in GBM exhibits context-dependent complexity. While SEMA3F suppresses U87 migration via the NRP2/PLXNA1 receptor complex [40], SEMA3A suppresses brain tumor stem cell proliferation but enhances migration through NRP1/PLXNA1 complex, and PLXNA1-knockdown reduced the growth of GBM in an mice model [41]. These findings suggest a potential protective role of PLXNA1 in certain contexts. Paradoxically, both NRP1 and PLXNA1 expressions correlate with poor clinical prognosis in GBM [42]. Our survival analyses using TCGA GBM datasets also reveal the worse outcomes in GBM patients with elevated PLXNA1 expression. This functional duality may stem from PLXNA1’s ability to engage diverse ligands and form distinct co-receptor complexes, thereby activating antagonistic downstream signaling cascades.

In the present study, we reveal that SEMA3G sensing leads to the activation of the intracellular GAP domain of PLXNA1 in GSCs, which in turn catalyzes the inactivation of Cdc42. As a key member of the Rho-GTPase protein family, aberrant activation or upregulation of Cdc42 has been associated with the malignant progression of glioma through PI3K/AKT signaling [43, 44]. In GSCs, we find that activated Cdc42 has a relatively high affinity for WWP2 and prevents the interactions between WWP2 and c-Myc. In contrast, the inactivation of Cdc42 induced by SEMA3G is barely able to sequester WWP2. Once released, WWP2, which is an E3 ubiquitin ligase [45], is responsible for c-Myc ubiquitination upon SEMA3G stimulation, which ultimately results in suppression of GSCs stemness. Therefore, our study revealed a new biological function of Cdc42, which acts as a “guard” to retain WWP2. It is a novel function pattern of Cdc42 that is different from the classic role of GTPases. Consistently, pharmacological inhibition of Cdc42 markedly inhibited c-Myc and GSCs. Targeting the SEMA3G-NRP2/PLXNA1-Cdc42 signaling pathway may be a novel therapeutic approach for treating GBM.

c-Myc plays an essential role in maintaining the self-renewal potential of embryonic stem cells and some cancer stem cells, including GSCs [46–49]. However, the development of therapies that target c-Myc is challenging. Ubiquitination-mediated protein degradation has been proven to be an effective method for clearing oncoproteins [50, 51]. Multiple E3 ubiquitin ligases including Cullin3-KCTD2, FBXL14, and FBXW7 have been implicated in c-Myc proteasomal degradation [52–54]. However, their structural complexity and limited ligand-binding pockets have proven challenging to be targeted pharmacologically [54]. In this study, we mechanistically identified WWP2 as the principal E3 ligase responsible for SEMA3G-triggered c-Myc ubiquitination and degradation. Intriguingly, neither dominant-negative Cdc42 expression nor WWP2 overexpression fully restored c-Myc protein levels upon SEMA3G treatment, suggesting additional compensatory pathways may contribute to this regulatory axis. While further elucidation of its interactome is required, our findings display SEMA3G-mediated c-Myc degradation as a promising strategy to circumvent the “undruggable” nature of GBM.

In conclusion, ECs-derived SEMA3G disrupts GSCs stemness via engagement with NRP2/PLXNA1 receptors, leading to the WWP2 mediated degradation of c-Myc. Genetic gain of *SEMA3G* or the recombinant SEMA3G protein significantly improved the survival of GBM-bearing mice. Hence, our work establishes a new paradigm for SEMA3G-mediated ECs-GSCs communication, which will help in the development of promising therapeutic strategies to improve the treatment of this highly lethal brain tumor.

## Supplementary information

Supplementary information

Original western blots

## Data Availability

Data supporting the findings of this study are available from the corresponding authors upon reasonable request.
