# Supplementary material for: Endothelial cells-derived SEMA3G suppresses glioblastoma stem cells by inducing c-Myc degradation
Source: Cell Death Differ. 2025 Jun 18;32(12):2340–54. doi: 10.1038/s41418-025-01534-3 (PMC12669739; doi:10.1038/s41418-025-01534-3)
Supplement: Supplementary file 1 — Supplementary information [file 41418_2025_1534_MOESM1_ESM.docx]

**Supplementary Information**

**Endothelial cells-derived SEMA3G suppresses glioblastoma stem cells by inducing c-Myc degradation**

Peng-Xiang Min^1,2#^, Li-Li Feng^3#^, Yi-Xuan Zhang^3^, [Chen-Chen Jiang](https://pubmed.ncbi.nlm.nih.gov/?term=Jiang+CC&cauthor_id=35817793)^3^, Hong-Zhen Zhang^3^, Yan Chen^1^, Kohji Fukunaga^4^, Fang Liu^2^，Yu-Jie Zhang^1^, Takuya Sasaki^5^, Xu Qian^6^, Katsuhisa Horimoto^7,8^, Jian-Dong Jiang^3,9^*, Ying-Mei Lu^1,10^*, Feng Han^3,11,12^*

^#^These authors contributed equally to this work.

*Correspondence authors Email: fenghan169@njmu.edu.cn (Feng Han)

lufx@njmu.edu.cn (Ying-Mei Lu), [jiang.jdong@163.com](mailto:jiang.jdong@163.com) (Jian-Dong Jiang)

Supplemental Figures and Table：

1. Supplemental Figures 1 to 10 and their legends

2. Supplemental Tables 1 to 3


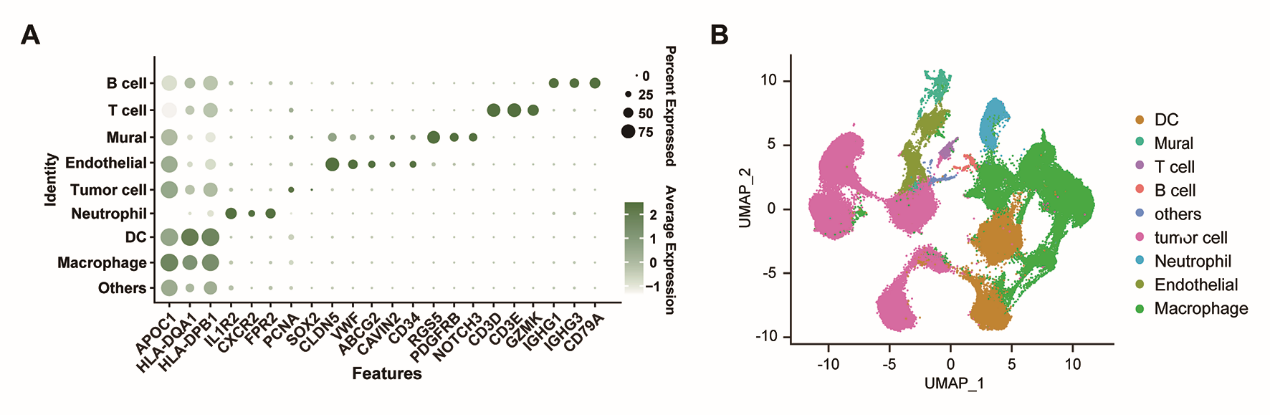


**Supplementary Fig. 1 scRNA-seq and cell type identification of GBM tissue. A** Dot plot heatmap of the marker genes in individual clusters (GSE162631). **B** Clustering of 46102 human GBM single-nuclei expression profiles into nine populations (GSE162631).


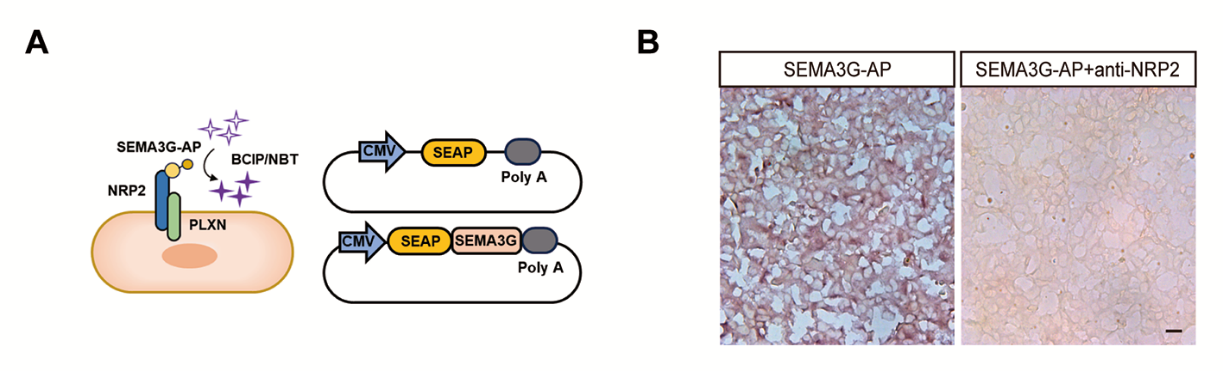


**Supplementary Fig. 2 SEMA3G binds to NRP2 in brain slices of GBM bearing mice. A** Schematic illustration of the alkaline phosphatase (AP)-tagged plasmid constructure and ligand binding experiment. **B** BCIP/NBT color development revealed the attenuated AP-SEMA3G binding in NRP2 neutralizing antibody treated brain sections of tumor-bearing mice. Scale bar, 5 μm. (n = 3).


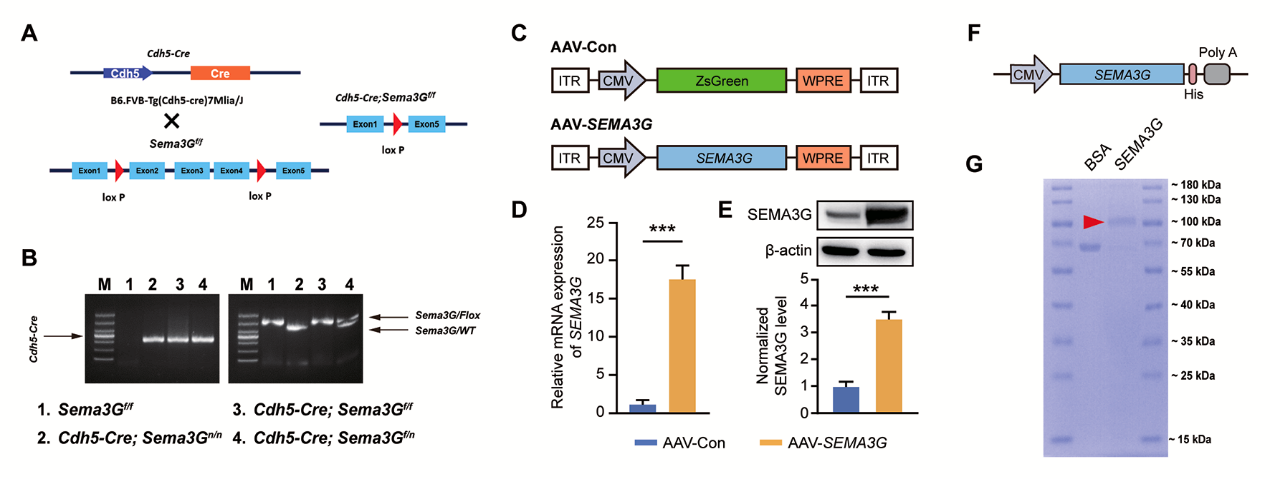


**Supplementary Fig. 3 Generation of *SEMA3G* gene manipulation and the efficiency identification. A** Schematic illustration of the generation of *Sema3G* endothelial cell conditional knockout mice (*Cdh5-Cre; Sema3G^f/f^* mice). **B** The genotype of *Cdh5-Cre; Sema3G^f/f^* mice were detected by PCR (n = 3). **C** Schematic diagram of the AAV used for SEMA3G overexpression *in vivo*. **D** The mRNA level of SEMA3G expression in nude mouse brain tissues at 14 days post-AAV injection using qPCR analysis. (n = 3). Data are shown as mean ± s.e.m. ^***^*P* < 0.001. Data were analyzed by two-tailed unpaired *t*-test. **E** The representative bands and quantification of the protein levels of SEMA3G in brain of nude mice treated as in (D), as determined by western blot analysis. Data are shown as mean ± s.e.m. (n = 3). ^***^*P* < 0.001. Data were analyzed by two-tailed unpaired *t*-test. **F** Schematic diagram of the plasmid used for purify human recombinant SEMA3G protein. **G** Coomassie staining of purified human recombinant SEMA3G protein.


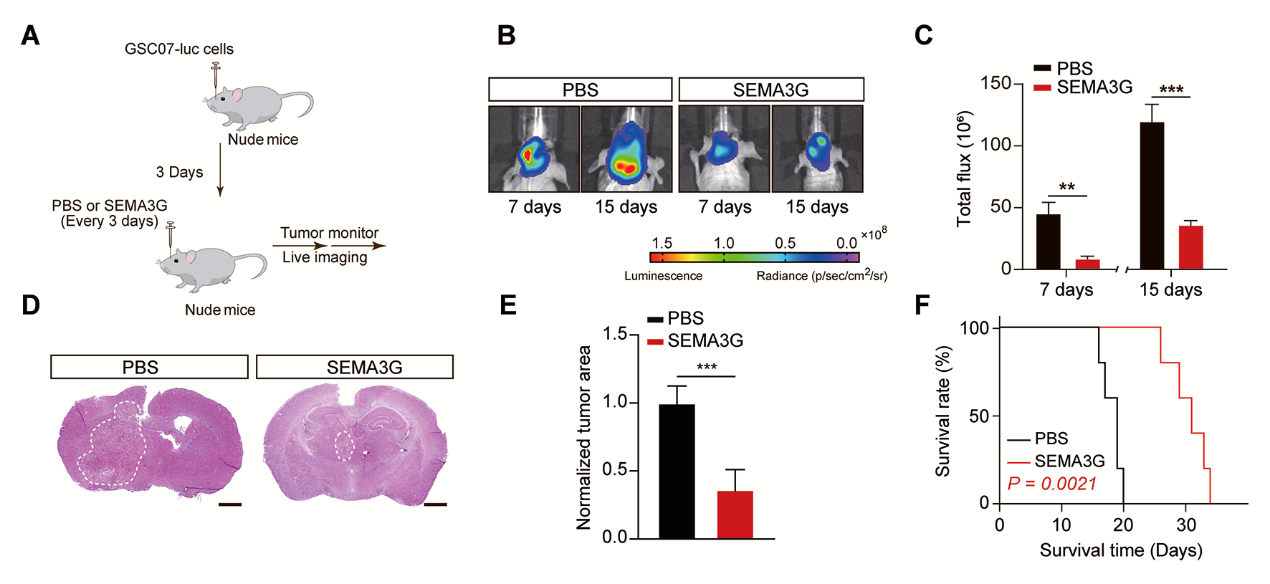


**Supplementary Fig. 4 Recombinant SEMA3G protein suppresses the *in vivo* GBM growth. A** Schematic diagram of recombinant SEMA3G protein (1 µg/mouse) or phosphate-buffered saline (PBS) in nude mice and the construction of GBM model using GSC07-Luc cells. **B, C** In vivo bioluminescent image (B) and quantification (C) of tumor growth in mice bearing heterologous GBM tissue on days 7 and 15 post-cells implant. Data are shown as mean ± s.e.m. n = 5, ***P* < 0.01, ****P* < 0.001. Data were analyzed by two-tailed unpaired *t*-test. **D** H&E staining of mouse brains collected 15 days post-GSC07-Luc cells transplantation. Scale bar, 1 mm. n = 5. **E** The tumor-to-brain area ratio was quantified from the H&E staining (D) using ImageJ software. Data are shown as mean ± s.e.m. n = 5, ****P* < 0.001, as determined by two-tailed unpaired *t*-test. **F** Kaplan-Meier survival curves of the tumor bearing nude mice. n = 5. The *P* values were calculated by the log-rank test. n = 5.


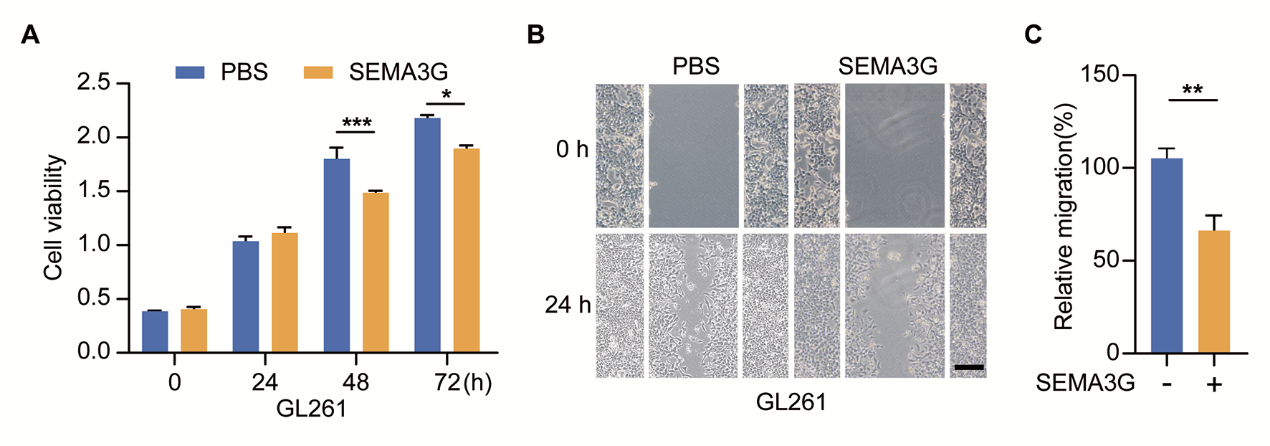


**Supplementary Fig. 5 SEMA3G inhibits GL261 growth and motility.** **A** GL261 cells were treated with recombinant SEMA3G protein (200 ng/ml) for the indicated days. Cell viability was determined by CCK8. Data are shown as mean ± s.e.m. ^*^*P* < 0.05, ^***^*P* < 0.001. Data were analyzed by one-way ANOVA. **B, C** GL261 cells were treated with recombinant SEMA3G protein (200 ng/ml) for 48 h, followed by a scratch. The cells were then maintained in the presence of SEMA3G for another 24 h and the cell motility was assessed by microscope (B) and quantified (C). Scale bar, 100 μm. ^**^*P* < 0.01, as determined by two-tailed unpaired *t*-test. All data collected from three independent experiments.


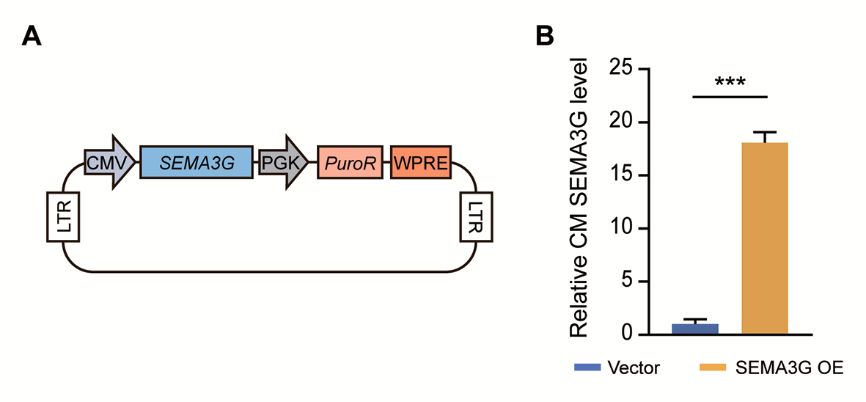


**Supplementary Fig. 6 Construction and identification of the stable SEMA3G overexpressing HUVEC cell line. A** Schematic diagram of the lentiviral plasmid used for SEMA3G overexpression in HUVECs. **B** The SEMA3G levels in supernatants of SEMA3G overexpressed HUVEC detected by ELISA. Data are shown as means ± s.e.m (n=3). Statistical analyses were determined by two-tailed unpaired *t*-test (B). ^***^*P* < 0.001.


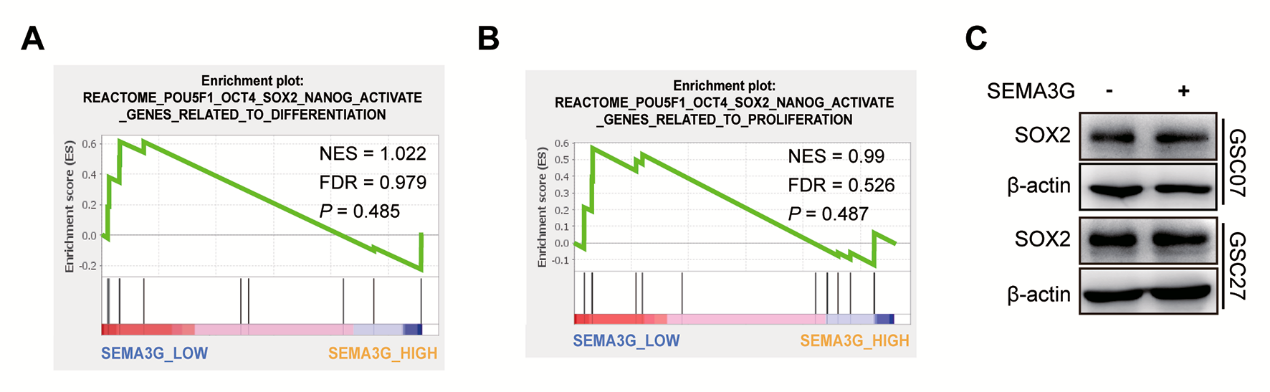


**Supplementary Fig. 7 SEMA3G barely influence the expression of SOX2.** **A, B** Gene set enrichment analysis comparing SOX2-related differentiation (A) and proliferation (B) gene sets in GBM samples categorized by SEMA3G expression levels (SEMA3G_LOW vs. SEMA3G_HIGH). **C** Representative band of SOX2 in GSC07 and GSC27 cells treated with or without recombinant hSEMA3G (200 ng/ml) for 72 h.


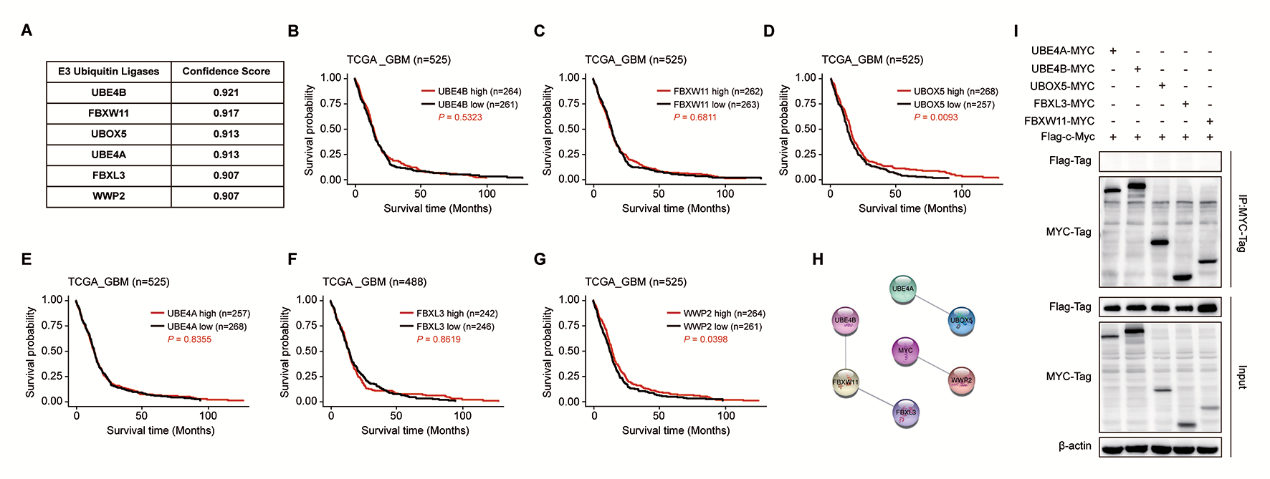


**Supplementary Fig. 8 WWP2 is an E3 ubiquitin ligase targeting c-Myc.** **A** UbiBrowser predicts c-Myc E3 ubiquitin ligase. **B-G** Kaplan-Meier survival analysis of UBE4B (B), FBXW11 (C), UBOX5 (D), UBE4A (E), FBXL3 (F) and WWP2 (G) in TCGA GBM datasets. The *P* values were calculated by the log-rank test. **H** c-Myc protein-protein interaction network (STRING-db.org). **I** Co-immunoprecipitation of c-Myc with UBE4A, UBE4B, UBOX5, FXBL3 and FXBW11 in HEK-293 cells transfected with Flag-tagged c-Myc and/or MYC-tagged UBE4A, UBE4B, UBOX5, FXBL3 and FXBW11 for 48 h. The western blot bands represent one of three independent experiments.


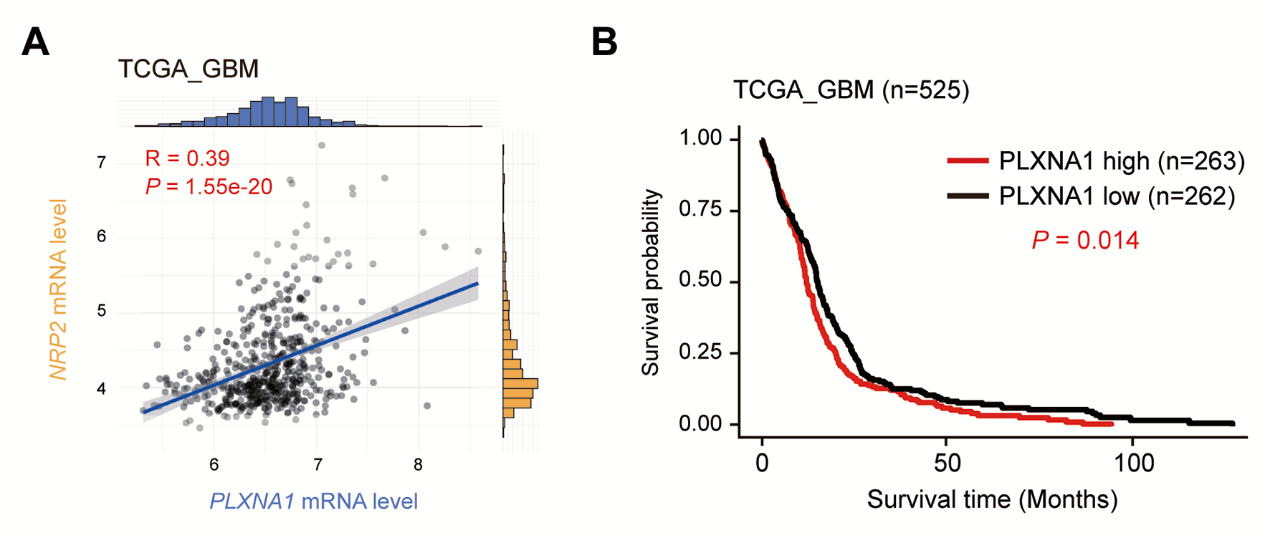


**Supplementary Fig. 9 PLXNA1 acts as a co-receptor for NRP2 in GBM. A** Correlation of NRP2 and PLXNA1 in GBM specimens from the TCGA GBM datasets was determined by Pearson’s correlation analysis. **B** Kaplan-Meier survival analysis of PLXNA1 in TCGA GBM datasets. The *P* values were calculated by the log-rank test.


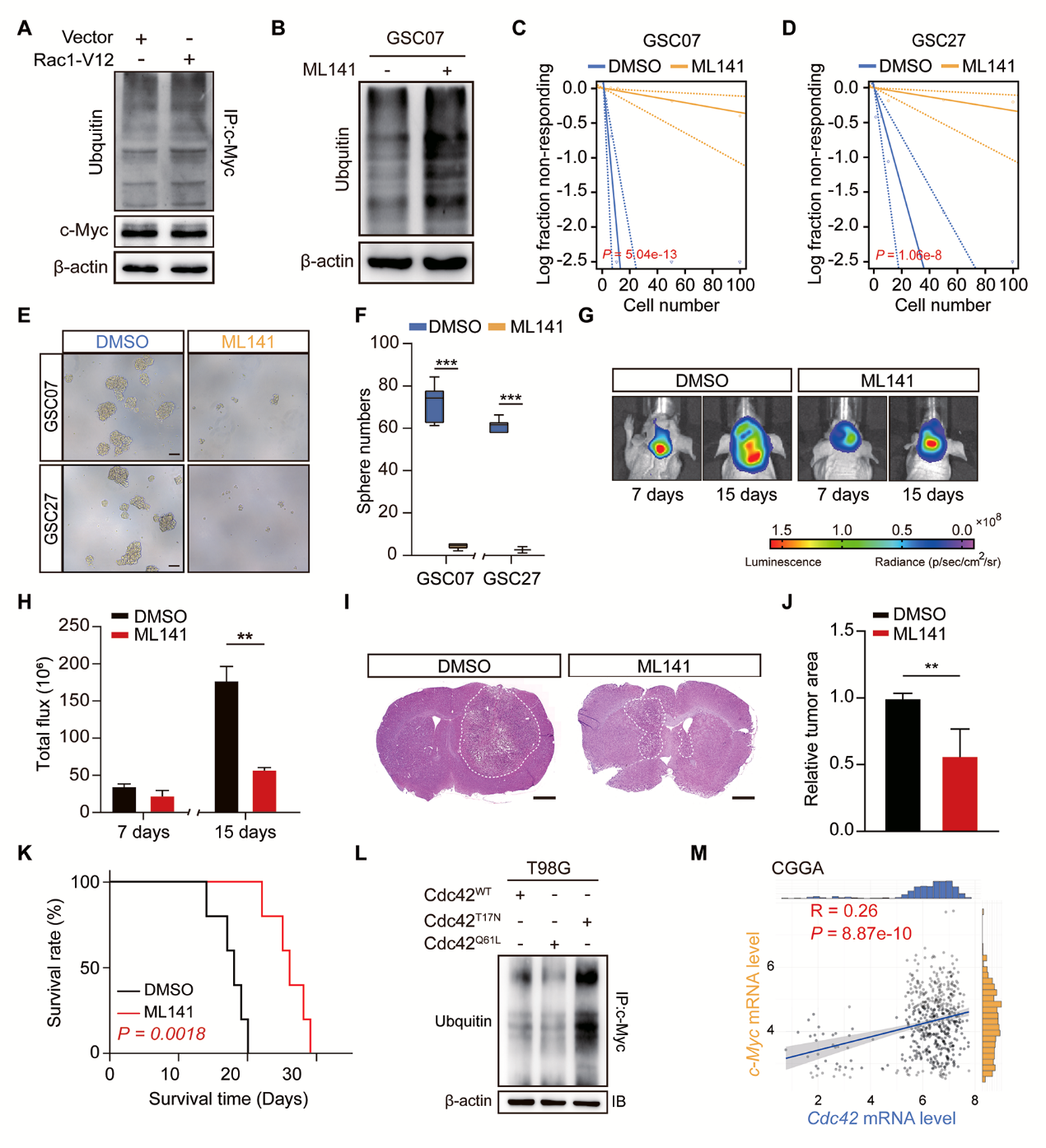


**Supplementary Fig. 10 Cdc42 serves as a downstream effector molecule in the SEMA3G signaling pathway.** **A** The representative bands of the ubiquitination level of c-Myc in T98G cells that transfected with GFP Tagged Rac1-V12 plasmids for 48 h. **B** The representative bands of the ubiquitination level in GSC07 cells treated with or without ML141 (10 μmol/l) for 24 h. **C, D** Sphere formation in GSC07 (C) and GSC27 (D) cells treated with or without ML141 (10 μmol/l) for 7 days (n = 5). **E, F** Representative images (E) and the number (F) of neurospheres of GSC07 and GSC27 treated with DMSO or ML141 for 7 days. Data are shown as mean ± s.e.m (n = 5). ^***^*P* < 0.001. Data were analyzed by two-way ANOVA. Scale bar, 100 μm. **G, H** Representative bioluminescent images (G) and quantification (H) of tumor growth in mice bearing heterotopic GBM xenografts on days 7 and 15 post-cells implant. Data are shown as mean ± s.e.m (n = 5). ^**^*P* < 0.01. Data was analyzed by two-tailed unpaired *t*-test. **I, J** Representative H & E staining of mouse brain sections (I) and the tumor-to-brain area ratio (J) in mice bearing GBM xenografts 15 days post-cell implant. Data are shown as mean ± s.e.m (n = 5). ^**^*P* < 0.01. Data were analyzed by two-tailed unpaired *t*-test. Scale bar, 1 mm. **K** Kaplan-Meier survival curves of tumor-bearing nude mice treated with DMSO or ML141 (n = 5). **L** The representative bands of the ubiquitination level of c-Myc in T98G cells transfected with GFP Tagged Cdc42^WT^, Cdc42^T17N^ or Cdc42^Q61L^ plasmids for 48 h. **M** Correlation of *Cdc42* and c-Myc at the transcriptional level from the CGGA datasets was determined by Pearson’s correlation analysis. All western blot bands represent one of three independent experiments.

Table S1. DNA oligos for shRNA

| Gene | Site | Sequence (5’->3’) |
| --- | --- | --- |
| WWP2 | #1 | CCCAAGGTGCATAATCGTCAA |
|  | #2 | CTCACCTACTTTCGCTTTATA |
| PLXNA1 | / | GCAGUACUGACAACGUCAATT |
| Negative control | / | UUCUCCGAACGUGUCACGUTT |

Table S2. Primer sets used for plasmid construction

| Gene | Standard | Sequence (5’->3’) |
| --- | --- | --- |
| Flag-c-Myc | Forward | CGAATTCATGCCCCTCAACGTTAGCTTC |
|  | Reverse | GCTCTAGATTACGCACAAGAGTTCCG |
| Flag-c-Myc  (1-354 aa) | Forward | CGAATTCATGCCCCTCAACGTTAGCTTC |
|  | Reverse | GCTCTAGATTAGACATTCTCCTCGGTGTCC |
| Flag-c-Myc  (145-354 aa) | Forward | CGAATTCATGGTCTCAGAGAAGCTGGCC |
|  | Reverse | GCTCTAGATTAGACATTCTCCTCGGTGTCC |
| Flag-c-Myc  (354-439 aa) | Forward | CGAATTCATGAAGAGGCGAACACACAACG |
|  | Reverse | GCTCTAGATTACGCACAAGAGTTCCG |
| c-Myc-VN173 | Forward | CGGAATTCCATGCCCCTCAACGTTAGC |
|  | Reverse | GCTCTAGACGCACAAGAGTTCCGTAGC |
| WWP2-MYC | Forward | CGGGATCCATGGCATCTGCCAGCTCTAGCCGGG |
|  | Reverse | GCTCTAGACTCCTGTCCAAAGCCCTCGGTCTCC |
| WWP2 OE | Forward | GGAATTCATGGCATCTGCCAGCTCTAG |
|  | Reverse | CGCGGATCCTTACTCCTGTCCAAAGCC |
| WWP2-VC155 | Forward | CGGAATTCCCATGGCATCTGCCAGCTCTAG |
|  | Reverse | GGGGTACCCTCCTGTCCAAAGCCCTCGG |
| NRP2-HA | Forward | CGGGGTACCATGGATATGTTTCCTCTCACCTGGG |
|  | Reverse | GCTCTAGATGCCTCGGAGCAGCACTTTTGGTGG |
| NRP2-TurboID | Forward | GCAAGCTTATGGATATGTTTCCTCTCACCTGG |
|  | Reverse | GCGAATTCTGCCTCGGAGCAGCACTTT |
| PLXNA1-MYC | Forward | CGGGGTACCATGCCGCTGCCACCGCGGAGC |
|  | Reverse | CGTCTAGAGCTGCTCAGGGCCATCGTGTC |
| Cdc42-GFP | Forward | GAAGATCTATGCAGACAATTAAGTGTG |
|  | Reverse | CGGGGTACCCCTAGCAGCACACACCTGCG |
| Cdc42^Q61L^-GFP | Forward | CTTTTTGATACTGCAGGGCTAGAGGATTATGACAGATTA |
|  | Reverse | ATCTGTCATAATCCTCTAGCCCTGCAGTATCAAAAAG |
| Cdc42^T17N^-GFP | Forward | GGGCGATGGTGCTGTTGGTAAAAATTGTCTCCTGAT |
|  | Reverse | ATATCAGGAGACAATTTTTACCAACAGCACCATCGC |
| Cdc42-CrN173 | Forward | CGGAATTCCATGCAGACAATTAAGTGTGTTG |
|  | Reverse | GCTCTAGATAGCAGCACACACCTGCGGC |
| Cdc42^Q61L^-CrN173 | Forward | CGGAATTCCATGCAGACAATTAAGTGTGTTG |
|  | Reverse | GCTCTAGATAGCAGCACACACCTGCGGC |
| Cdc42^T17N^-CrN173 | Forward | CGGAATTCCATGCAGACAATTAAGTGTGTTG |
|  | Reverse | GCTCTAGATAGCAGCACACACCTGCGGC |
| SEMA3G-AP | Forward | GCTCTAGAATCATCCCAGTTGAGGAGG |
|  | Reverse | GCTCTAGATCAGTCGGTGGTGCCGGCG |
| UBE4A-MYC | Forward  Reverse | CGGGATCCATGACAGACCAGGAGAAT  GCTCTAGATTCAAGTTGCTCCTTTTGTT |
| UBE4B-MYC | Forward | CGGGATCCATGGAGGAGCTGAGCGCT |
|  | Reverse | GCTCTAGAGTGATCGCTGTTCTGTTTCT |
| UBOX5-MYC | Forward | CGGGATCCATGGTAATAAATCTTTGCCTC |
|  | Reverse | GCTCTAGAGAAGTGGACCCGCAGCACGT |
| FBXL3-MYC | Forward | CGGGATCCATGAAACGAGGAGGAAGAGA |
|  | Reverse | GCTCTAGACCAAGTGGGCATCATGTC |
| FBXW11-MYC | Forward | CGGGATCCATGGAGCCCGACTCGGTGAT |
|  | Reverse | GCTCTAGATCTAGAGATGTAAGTGTATGT |
| Rac1-V12-GFP | Forward | AGTTTTACCTACAGCTACGTCTCCCACCACCAC |
|  | Reverse | GTGGTGGTGGGAGACGTAGCTGTAGGTAAAACT |
| SEMA3G OE | Forward | CGGAATTCATGGCCCCCTCGGCCTG |
|  | Reverse | GCTCTAGACTACGTGGCCTCCAC |
| SEMA3G-His | Forward | GGGGTACCATGGCCCCCTCGGCCTG |
|  | Reverse | CGGAATTCCGTGGCCTCCACCTCCCG |

Table S3. Primer sets used for quantitative real-time PCR

| Gene | Standard | Sequence (5’->3’) |
| --- | --- | --- |
| SEMA3G | Forward | CTCAAAGTCATCGCTCTCCAGGC |
|  | Reverse | AGTGCCGTAAGTCTCACATTGG |
| c-Myc | Forward | GTCAAGAGGCGAACACACAAC |
|  | Reverse | TTGGACGGACAGGATGTATGC |
| CCNA2 | Forward | GGATGGTAGTTTTGAGTCACCAC |
|  | Reverse | CACGAGGATAGCTCTCATACTGT |
| CDC25A | Forward | GATGATGGCTTCGTGGACCTTCTC |
|  | Reverse | ACTGACCGAGTGCTGGAGCTAC |
| CDK4 | Forward | GGGGACCTAGAGCAACTTACT |
|  | Reverse | CAGCGCAGTCCTTCCAAAT |
| PCNA | Forward | TCGTCCCACGTCTCTTTGGT |
|  | Reverse | ATCTTCATTGCCGGCGCATT |
| EIF4E | Forward | ACTGTCGAACCGGAAACCA |
|  | Reverse | CAAACTTGGAGATCAGCCGC |
| CDKN1A | Forward | CAGACCAGCATGACAGATTTCTAC |
|  | Reverse | AAGGCAGAAGATGTAGAGCGG |
| CASP3 | Forward | TGGAAGCGAATCAATGGACTCTGG |
|  | Reverse | CAGACCGAGATGTCATTCCAGTGC |
| GADD45A | Forward | TGCGAGAACGACATCAACATCCTG |
|  | Reverse | TGAATGTGGATTCGTCACCAGCAC |
| ACTB | Forward | TGGTGATGGAGGAGGTTTAGTAAGT |
|  | Reverse | AACCAATAAAACCTACTCCTCCCTTAA |
