# Supplementary material for: Endothelial cells-derived SEMA3G suppresses glioblastoma stem cells by inducing c-Myc degradation
Source: Cell Death Differ. 2025 Jun 18;32(12):2340–54. doi: 10.1038/s41418-025-01534-3 (PMC12669739; doi:10.1038/s41418-025-01534-3)

Fig1N

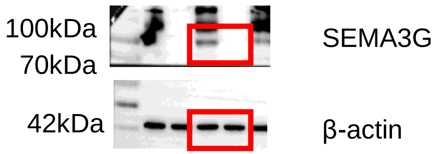


Fig4D

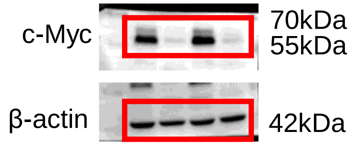


Fig4H

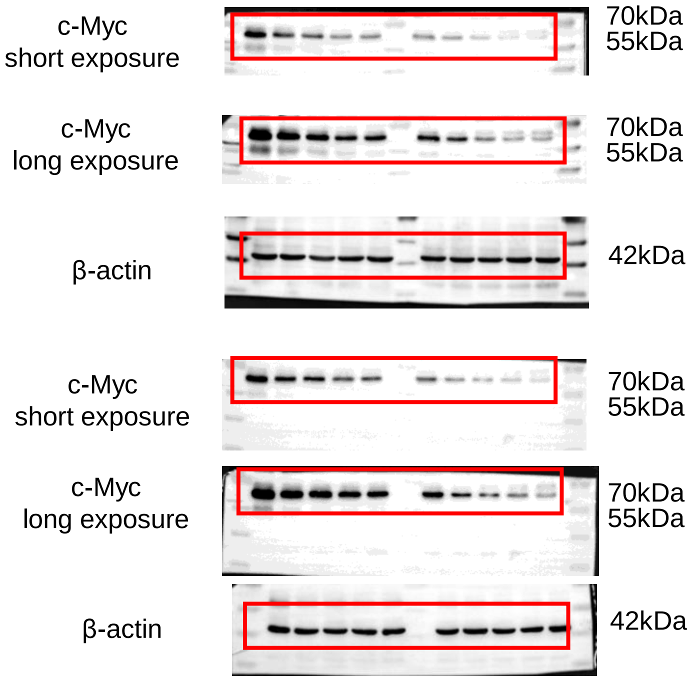


Fig4L

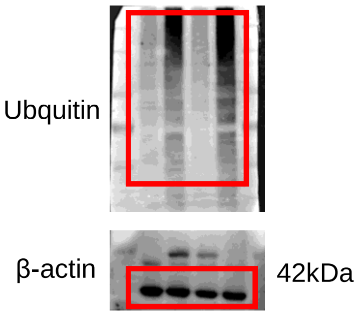


Fig4M

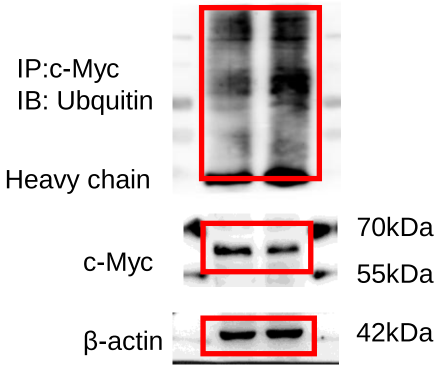


Fig4O

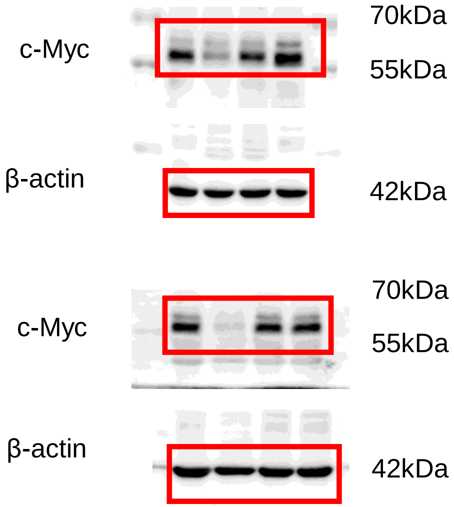

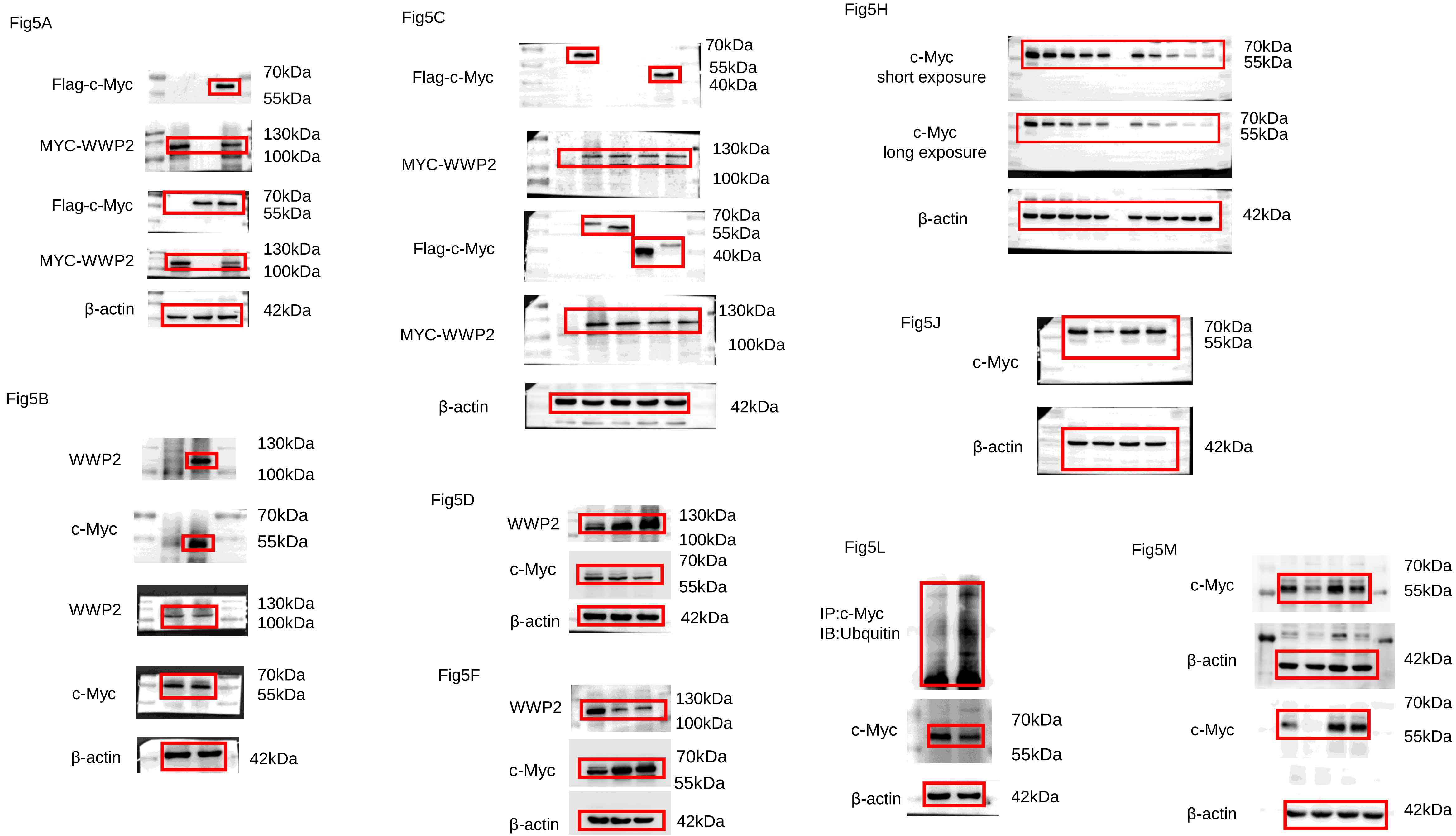
Fig5A
Flag-c-Myc
70kDa
55kDa
MYC-WWP2
130kDa
100kDa
Flag-c-Myc
70kDa
55kDa
MYC-WWP2
130kDa
100kDa
β-actin
42kDa
Fig5B
WWP2
130kDa
100kDa
c-Myc
70kDa
55kDa
WWP2
130kDa
100kDa
c-Myc
70kDa
55kDa
β-actin
42kDa
Fig5C
Flag-c-Myc
70kDa
55kDa
40kDa
MYC-WWP2
130kDa
100kDa
Flag-c-Myc
70kDa
55kDa
40kDa
MYC-WWP2
130kDa
100kDa
β-actin
42kDa
Fig5D
WWP2
130kDa
100kDa
c-Myc
70kDa
55kDa
β-actin
42kDa
Fig5F
WWP2
130kDa
100kDa
c-Myc
70kDa
55kDa
β-actin
42kDa
Fig5H
c-Myc
short exposure
70kDa
55kDa
c-Myc
long exposure
70kDa
55kDa
β-actin
42kDa
Fig5J
c-Myc
70kDa
55kDa
β-actin
42kDa
Fig5L
IP:c-Myc
IB:Ubquitin
c-Myc
70kDa
55kDa
β-actin
42kDa
Fig5M
c-Myc
70kDa
55kDa
β-actin
42kDa
c-Myc
70kDa
55kDa
β-actin
42kDa

Fig6B

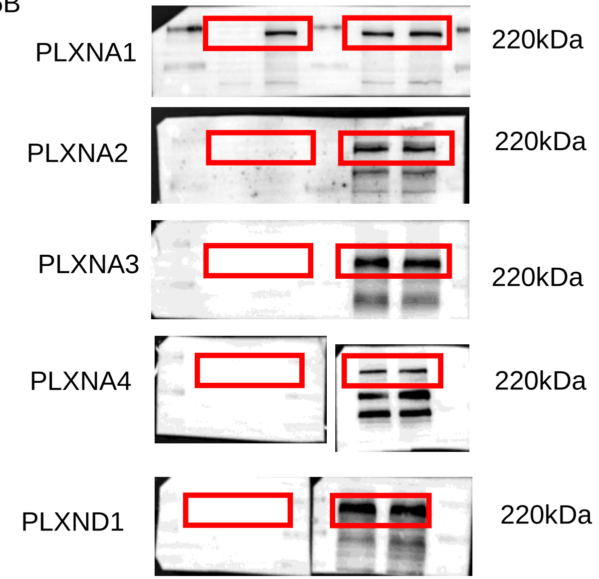


Fig6C

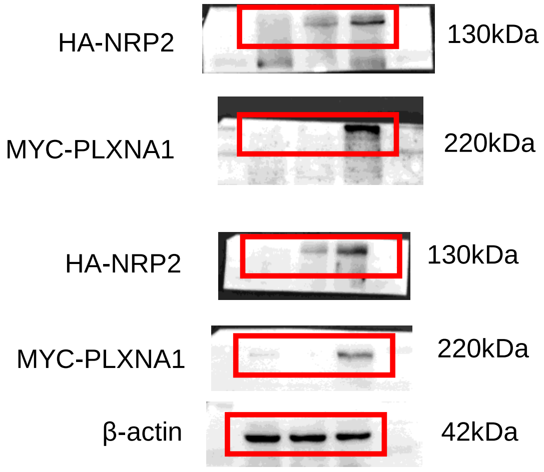


Fig6D

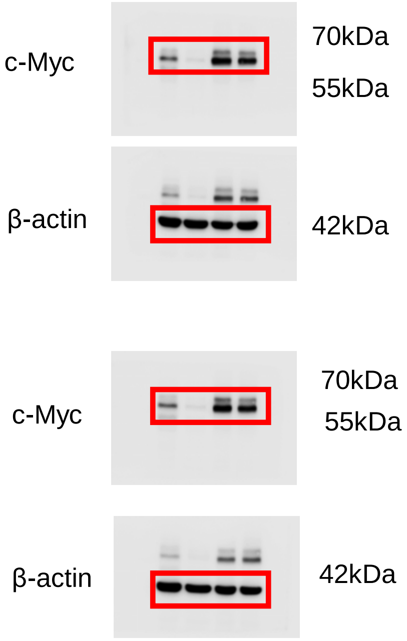


Fig6J

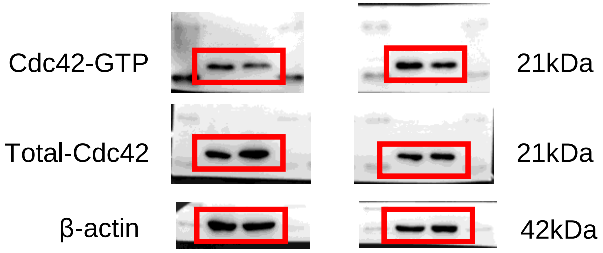


Fig6L

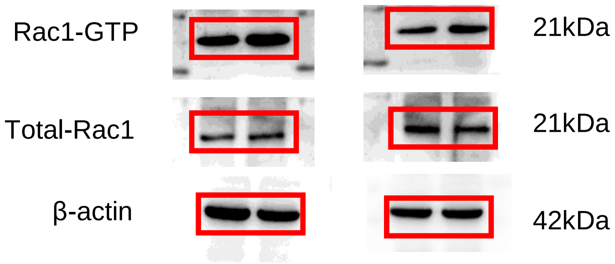


Fig6N

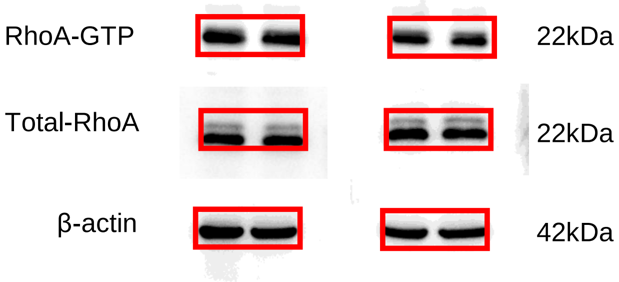

Fig7A

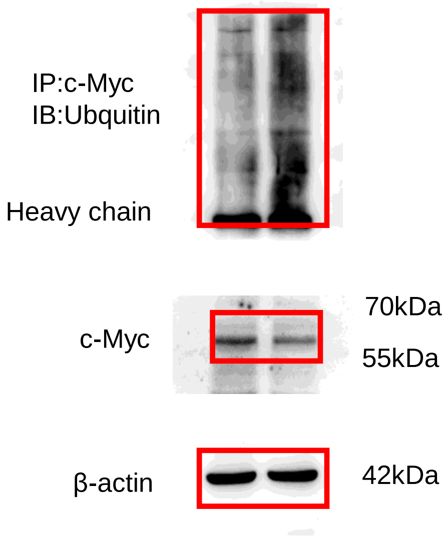


Fig7B

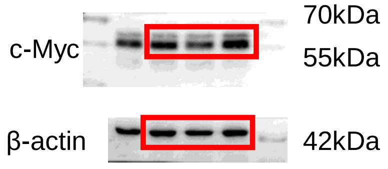


Fig7G

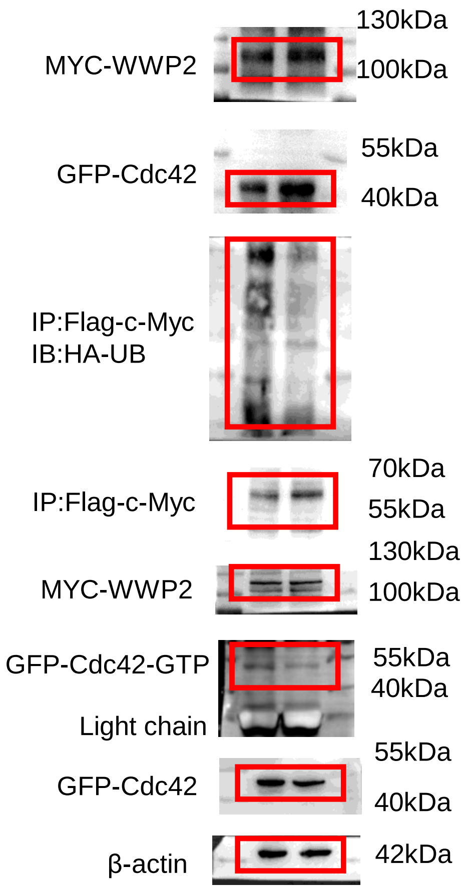


Fig7J

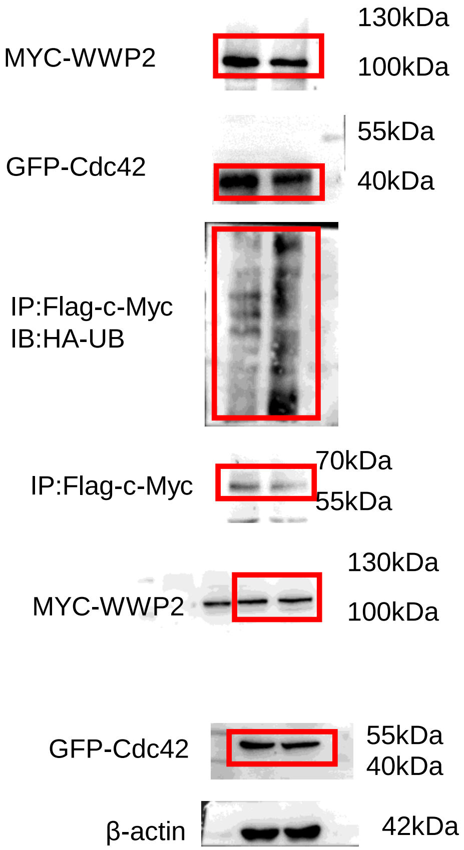


Fig7K

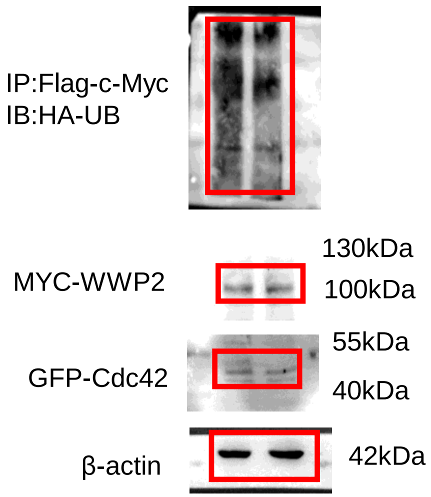

FigS3E

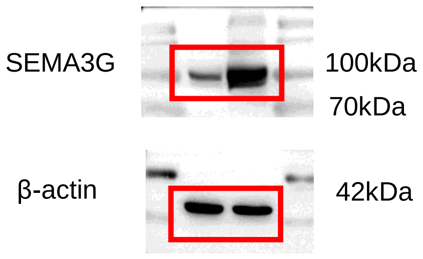


FigS7O

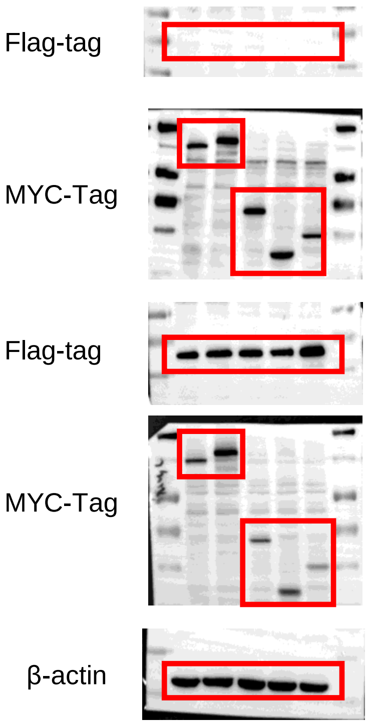


FigS10A

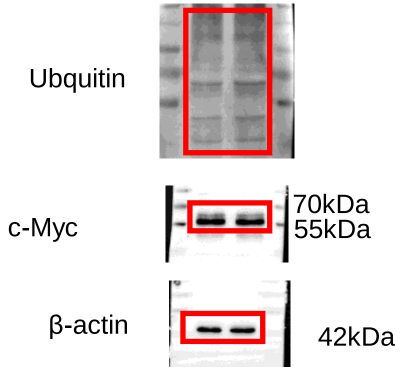


FigS10L

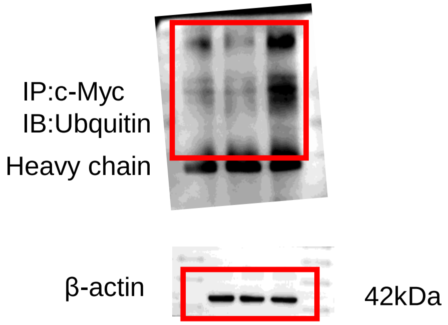


FigS6C

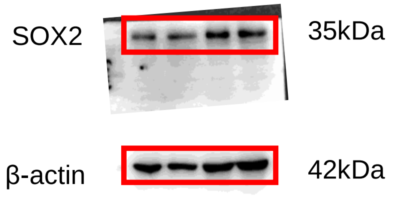


FigS10B

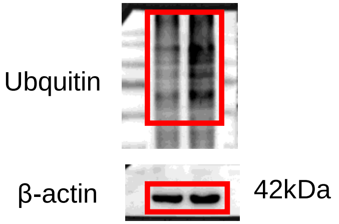

Supplement: Supplementary file 2 — Original western blots [file 41418_2025_1534_MOESM2_ESM.pdf]
